# A real-world pharmacovigilance study on cardiovascular adverse events of tisagenlecleucel using machine learning approach

**DOI:** 10.1038/s41598-024-64466-x

**Published:** 2024-06-13

**Authors:** Juhong Jung, Ju Hwan Kim, Ji-Hwan Bae, Simon S. Woo, Hyesung Lee, Ju-Young Shin

**Affiliations:** 1 Department of Biohealth Regulatory Science, Sungkyunkwan University, Suwon, Republic of Korea; 2 School of Pharmacy, Sungkyunkwan University, 2066, Seobu-ro, Jangan-gu, Suwon, Gyeonggi-do 16419 Republic of Korea; 3 Department of Artificial Intelligence, College of Computing and Informatics, Sungkyunkwan University, Suwon, Republic of Korea; 4 Samsung Advanced Institute for Health Sciences & Technology, Sungkyunkwan University, Seoul, Republic of Korea

**Keywords:** Chimeric antigen receptor T-cell, Cardiotoxicity, Machine learning algorithm, Pharmacovigilance, Cardiovascular diseases, Data mining, Machine learning, Medical research

## Abstract

Chimeric antigen receptor T-cell (CAR-T) therapies are a paradigm-shifting therapeutic in patients with hematological malignancies. However, some concerns remain that they may cause serious cardiovascular adverse events (AEs), for which data are scarce. In this study, gradient boosting machine algorithm-based model was fitted to identify safety signals of serious cardiovascular AEs reported for tisagenlecleucel in the World Health Organization Vigibase up until February 2024. Input dataset, comprised of positive and negative controls of tisagenlecleucel based on its labeling information and literature search, was used to train the model. Then, we implemented the model to calculate the predicted probability of serious cardiovascular AEs defined by preferred terms included in the important medical event list from European Medicine Agency. There were 467 distinct AEs from 3,280 safety cases reports for tisagenlecleucel, of which 363 (77.7%) were classified as positive controls, 66 (14.2%) as negative controls, and 37 (7.9%) as unknown AEs. The prediction model had area under the receiver operating characteristic curve of 0.76 in the test dataset application. Of the unknown AEs, six cardiovascular AEs were predicted as the safety signals: bradycardia (predicted probability 0.99), pleural effusion (0.98), pulseless electrical activity (0.89), cardiotoxicity (0.83), cardio-respiratory arrest (0.69), and acute myocardial infarction (0.58). Our findings underscore vigilant monitoring of acute cardiotoxicities with tisagenlecleucel therapy.

## Introduction

Chimeric antigen receptor T-cell (CAR-T) therapies have shown a new paradigm in patients affected by hematological malignancies including relapsed or refractory leukemias and lymphomas^1^. Tisagenlecleucel is one of the most widely used CD-19 directed CAR-T products. In JULIET, a phase 2 single-arm trial, patients with B-cell lymphoblastic leukemia had an overall response rate of 52% (complete response rate of 40%, partial response rate of 12%) after receiving tisagenlecleucel^2^. Moreover, in children and young adult patients with B-cell lymphoblastic leukemia, tisagenlecleucel also demonstrated promising remission and 1-year overall survival rates of 80% and 70%, respectively^3^.

With the recent increase in its use followed potential safety concerns that may necessitate risk–benefit assessment of CAR-T therapy^4^. These included a recent safety warning of serious risk of secondary malignancy released by the US Food and Drug Administration (FDA) and European Medicines Agency (EMA)^5,6^, and neurotoxicity associated with CAR-T therapy^7^. Moreover, adverse cardiovascular outcomes have been described among patients receiving CAR-T therapies^8–11^. Previous studies suggest that the interaction between melanoma-associated antigen-3 expressed by CAR-T and titin, one of the protein types in cardiac muscle, could cause fatal myocarditis or cardiogenic shock^12^. Furthermore, cytokine-associated cardiotoxicity could occur as a result of cytokine release syndrome (CRS), another well-known complication of CAR-T therapy^13,14^.

Few studies to date have systemically described the cardiovascular safety profiles of CAR-T therapies in the real-world population. In the post-marketing safety surveillance studies using the World Health Organization (WHO) pharmacovigilance database (VigiBase), CRS accounted for 66.4% of the adverse events (AEs) reported for CAR-T therapies, followed by wide ranges of cardiovascular AEs detected as the safety signals^15^. These findings were also consistently noted in the analysis of US FDA adverse event reporting system (FAERS) database, in which significant disproportionate reporting of cardiomyopathies was noted for axicabtagene ciloleucel (reporting odds ratio 2.3; 95% confidence interval 1.2–4.4), another commercially available CAR-T product^16^. These studies have provided a meaningful insight into the real-world safety of CAR-T therapy using disproportionality analysis method commonly utilized for screening safety signals in the drug safety surveillance database.

Despite the widely accepted use in drug safety surveillance, disproportionality analysis often shows modest accuracy as it simply relies on disproportionate reporting frequencies in detecting the safety signals without considering for other features available in the database^17,18^. Moreover, given that the analysis estimates relative rate of reporting of an AE, the safety signals may depend on the choice of the reference group (i.e., comparator drug)^19^. To overcome these limitations, machine learning has been implemented for detecting safety signals in the drug safety surveillance database^20^. Rather than relying on a two-by-two contingency table in estimating disproportionate reporting of an AE, machine learning utilizes all available features in a dataset to construct predictive model for safety signal detection. Of the machine learning methods that had been implemented in drug safety surveillance, two ensemble methods, gradient boosting machine (GBM) and random forest (RF) have demonstrated superior predictive performance in detecting new safety signals in the real-world data^21^. Our previous published works also have showed superior performance of GBM and RF over the traditional disproportionality analysis in detecting safety signals in spontaneous AE reporting database^22^, and GBM out-performing RF in predicting new safety signals of anti-cancer agents^23^.

In this regard, we conducted a pharmacovigilance study by utilizing a supervised machine learning, GBM, to identify serious cardiovascular AEs reported following CAR-T therapy with tisagenlecleucel in the VigiBase. We specifically focused on tisagenlecleucel as it is one of the first approved and most widely used among commercially available CAR-T products, which would ensure sufficient number of AE reports for machine learning training and model fitting.

## Methods

### Data source

This was an observational, retrospective, pharmacovigilance study using AEs reported in the WHO’s VigiBase. It contains over 30 million safety reports on AEs collected from more than 150 countries. These reports originate from various sources, including healthcare professionals, patients, and pharmaceutical companies. Each report contains information on the reporter’s qualification, patient information, drugs and suspected AEs. Drugs listed in the safety reports are recorded as “suspected” or “interacting” if potentially responsible for causing the AEs, or “concomitant” if not responsible, determined by the reporter. The AEs are coded according to the Medical Dictionary for Regulatory Activities (MedDRA) terms. The study data was obtained through “VigiBase Extract Case Level” service provided by WHO-Uppsala Monitoring Centre. This service delivers raw data as a fixed length text files that can be setup as a relational database using unique report identification numbers to join between the provided datasets. The extracted data used in this study contained individual case safety reports up to February 2024. All analysis was conducted using medDRA version 26.1.

### Data processing

From the total reports in VigiBase between January 1976 and February 2024, AE reports that listed tisagenlecleucel as a “suspected” or “interacting” drugs were included in the study analysis; other reports recorded as “concomitant” or missing suspected drug information were excluded. Then, we constructed label and feature data for training and fitting of a machine learning method.

Label data contains information on the known and unknown AEs of a drug of interest. Known AEs are used as orientation for training and testing of machine learning, and unknown AEs for mining new safety signals. In this study, the label data was constructed using the AEs recorded for tisagenlecleucel in the study data. Depending on whether those AEs were listed in the EMA product label or scientific literature, we categorized them into three groups: (1) AE associated with drug (i.e., positive control), if the relationship between drug and AE was described in randomized controlled trials (RCT), observational cohort studies, or listed in the product label; (2) AEs not associated with drug (i.e., negative control), if there is no available documents describing the relationship; (3) Unknown AEs (i.e., unknown), if the relationship was only described in case reports, case series or other relevant literature, but not listed in the product label information or described in the RCT or observational cohort studies. All AEs were identified using the preferred terms (PT) code of MedDRA.

Feature data represents variables recorded in the AE reports that is used for improving predictive performance of machine learning. Specifically, distributions of the variables such as reporter characteristics, demographics and frequency of reported cases for each AE are used for model fitting. In this study, of the variables available from the AE reports of tisagenlecleucel, we selected the following variables based on the European Medicine Agency (EMA) Guideline on good pharmacovigilance practices (GVP)–Module IX-signal Management^24^: number of cases, reactions after drug interruption, reactions after re-dosing drug, number of reporting by healthcare professional, seriousness of adverse events, and outcome of adverse events.

### End points

The primary endpoint was serious cardiovascular AEs classified as “unknown”. EMA Important medical event terms list was used to classify 11 serious cardiac AEs (pericardial effusion, cardiotoxicity, bradycardia, cardio-respiratory arrest, cardiorenal syndrome, pulseless electrical activity, arrhythmia, cardiomyopathy, cardiopulmonary failure, acute myocardial infarction, aortic valve incompetence). From the safety reports listing any of these AEs, we collected information on the reporter type, “serious AE” designation, indication for the suspected drug(s), age group (pediatrics, adults, elderly), and time-to-onset of the AE. Secondary endpoint was cardiovascular AEs co-reported with CRS. For this endpoint, we analyzed the safety reports that listed both CRS and the cardiovascular AEs with tisagenlecleucel.

### Modeling strategies

In the process of model construction, safety reports designated either as positive or negative controls were included to construct an input dataset, for which 75% of the dataset was used for model training and 25% for model testing. Then, we applied the established input dataset to a GBM algorithm-based model^25^. GBM is an algorithm with a boosting structure that is learned by reducing errors between predicted and actual data. Based on the error of randomly generated decision tree, a new decision tree is created in a direction of decreasing the model gradient, and decision trees are combined to create one optimal model. Specifically, we used the extreme gradient boosting (XGBoost) as a method for fitting the GBM algorithm. XGBoost has been shown to reduce overfitting of the algorithm by allowing a model to learn the boosting algorithm in parallel^26^. We generated and validated XGBoost-based model that calculates a probability of whether an AE (i.e., cardiovascular AE) is associated with a drug-of-interest (i.e., tisagenlecleucel) using the training dataset. Lastly, we implemented the model to calculate predicted probability of the cardiovascular AEs identified by PT codes included in important medical event lists from EMA. We determined that specific AE is associated with tisagenlecleucel if the predicted probability was greater than 0.5, which is a default decision threshold of a binary classifier. This was also a liberal prediction threshold which would enable for more safety signals to be detected and evaluated further for their association with the suspected drugs.

### Statistical analysis

Synthetic Minority Over-sampling Technique (SMOTE) was used to handle imbalance in the label data. SMOTE is one of the oversampling techniques for data processing, based on the k-NN algorithm^27^. To evaluate the performance of developed signal prediction model, we measured the area under the receiver operating characteristic curve (AUROC), accuracy and F1 score. AUROC $(=\frac{{Sensitivity+Specificity}}{{2}})$ represents an average precision, and with the value generally between 0.5 and 1, it determines that closer to 1, the more accurate the model predicts. F1 score$(=\frac{{2}}{{\frac{{1}}{{precision}}+\frac{{1}}{{Recall}}}})$ is a harmonic mean of precision and recall, and closer the value is to 1, the better performance of binary classification model. Accuracy is the proportion of data correctly predicted by the classification model among the total datasets. These performance indicators were selected and measured based on the guideline on GVP – Module IX – signal management^24^.

Furthermore, we compared the statistical performance (Accuracy, Sensitivity, Specificity, PPV, Negative predictive value, AUROC) in our prediction machine against the traditional signal detection methods including information component (IC), proportional reporting ratio (PRR), reporting odd ratio (ROR), empirical bayes geometric mean (EBGM).

All analyses were conducted using Python version 3.9.13 and SAS version 9.4. All methods used in this study were performed in accordance with the relevant guidelines and regulations.

### Ethics approval

The institutional review board of Sungkyunkwan University approved the study (IRB No. SKKU 2023-02-024); the board waived the requirement for obtaining informed consent as this study used anonymized administrative data.

## Results

### Characteristics of the AE case reports of tisagenlecleucel

**Figure 1:**
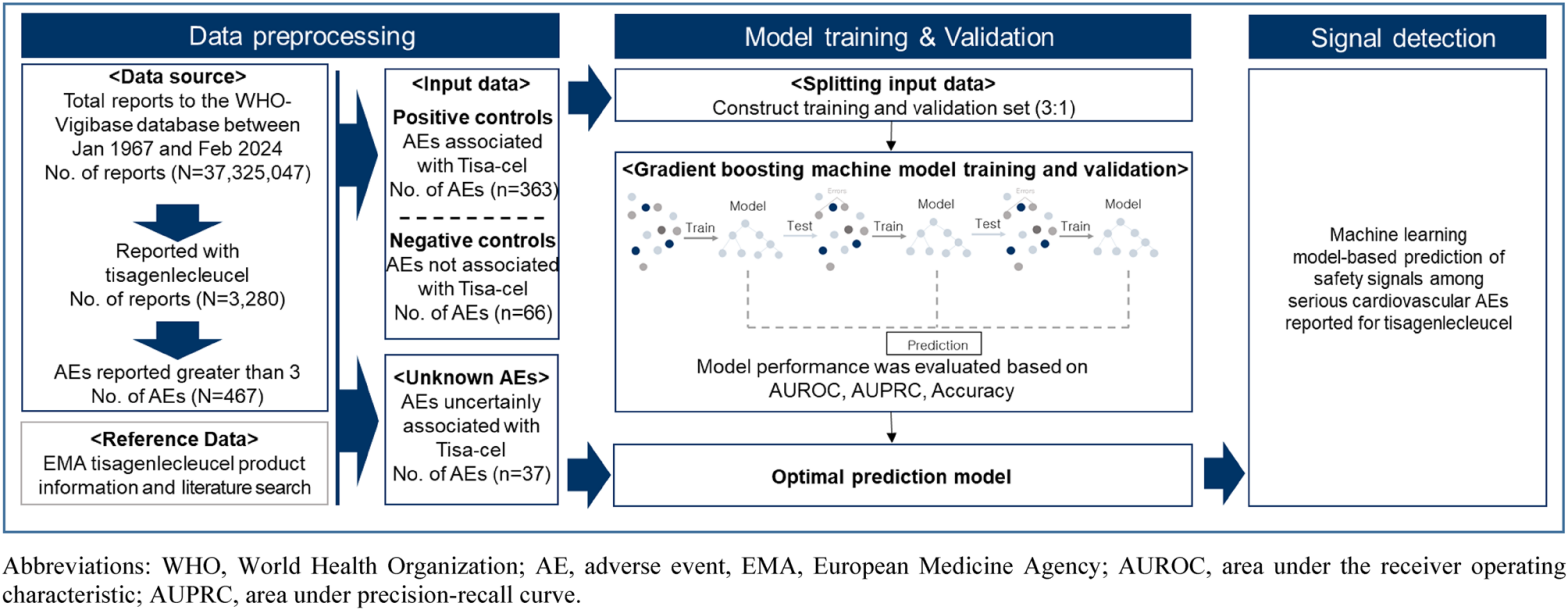
Study scheme of machine learning based signal detection.

Of 37.3 million reports recorded in VigiBase up until February 2024, we extracted and analyzed 3,280 safety reports that listed tisagenlecleucel. In these reports, there were 467 distinct AE terms (i.e., PT codes), of which 363 (77.7%) were positive control, 66 (14.1%) negative control, and 37 (7.9%) unknown AEs (Fig. 1 and Supplementary Table 1).

There were 72 reports of serious cardiovascular AEs, of which 43 (59.7%) resulted in death or fatal event and 15 (20.8%) in hospitalization. Most of them were adults aged 19–64 years (48.6%), followed by pediatrics (37.5%) and elderly (9.7%). The most frequently recorded indication for tisagenlecleucel was acute lymphoblastic leukemia (50.0%), followed by non-Hodgkin’s lymphoma (43.1%) (Table 1).

**Table 1:** Demographic characteristics of serious cardiovascular adverse event reports on tisagenlecleucel in WHO VigiBase up until February 2024.

Characteristics	Tisagenlecleucel (N, %)
Total no. of serious cardiovascular AE case reports	72 (100%)
Serious AE category	
Death/fatal	43 (59.7%)
Hospitalization	15 (20.8%)
Gender	
Male	40 (55.6%)
Female	28 (38.9%)
Reporter type	
Health professionals	65 (92.9%)
Indication for tisagenlecleucel	
Non-Hodgkin’s Lymphoma	31 (43.1%)
Acute Lymphoblastic Leukemia	36 (50.0%)
Age group	
Pediatrics	27 (37.5%)
Adults	35 (48.6%)
Elderly	7 (9.7%)
Serious cardiovascular AEs co-reported with cytokine release syndrome	
Bradycardia	12 / 17 (70.6%)
Pericardial effusion	11 / 15 (73.3%)
Cardio-respiratory arrest	7 / 8 (87.5%)
Pulseless electrical activity	4 / 8 (50.0%)
Cardiotoxicity	5 / 7 (71.4%)
Acute myocardial infarction	2 / 3 (66.6%)
Median (IQR) time to AE onset, days	9.5 (4.5–43)

*WHO* World Health Organization, *AE* adverse event, *IQR* Interquartile Range.

Median time-to-onset of serious cardiovascular AEs was 9.5 days (interquartile range 4.5–43 days). Serious cardiovascular AEs reported together with CRS included cardio-respiratory arrest (87.5%), pericardial effusion (73.3%), cardiotoxicity (71.4%), bradycardia (70.6%), acute myocardial infarction (66.6%) and pulseless electrical activity (50.0%) (Table 1).

### Serious cardiovascular AEs associated with tisagenlecleucel

Of 11 serious cardiovascular AEs, the GBM predicted six safety signals of tisagenlecleucel: bradycardia (17 cases; predicted probability 0.99), pericardial effusion (15; 0.98), cardio-respiratory syndrome (8; 0.69), pulseless electrical activity (8; 0.89), cardiotoxicity (7; 0.83), acute myocardial infarction (3; 0.58). Traditional disproportionality analyses also detected the most of the safety signals of GBM, except for acute myocardial infarction. Cardiorenal syndrome (8 cases; predicted probability 0.19) and cardiopulmonary failure (3; 0.25) that did not meet the signal threshold of GBM were detected as the safety signals in the disproportionality analyses (Table 2).

**Table 2:** Safety signals of serious cardiovascular adverse events reported with tisagenlecleucel in the WHO VigiBase up until February 2024.

Serious cardiac AEs^a^	No. of reports	Probability^b^ (threshold > 0.5)	PRR05^c^	ROR05^c^	IC05^c^	EBGM05^c^
Bradycardia	17	0.99	1.60	1.60	0.48	1.68
Pericardial effusion	15	0.98	5.44	5.43	1.97	5.23
Cardio-respiratory arrest	8	0.69	1.71	1.71	0.37	1.79
Pulseless electrical activity	8	0.89	15.95	15.94	2.29	16.07
Cardiorenal syndrome	8	0.19	161.11	160.97	2.80	145.14
Cardiotoxicity	7	0.83	10.59	10.59	1.90	9.63
Cardiomyopathy	4	0.07	1.29	1.29	−0.33	1.37
Acute myocardial infarction	3	0.58	0.48	0.48	−1.60	0.59
Aortic valve incompetence	3	0.10	1.96	1.96	−0.25	1.79
Arrhythmia	3	0.03	0.13	0.13	−3.21	0.18
Cardiopulmonary failure	3	0.25	5.28	5.28	0.29	3.85

*WHO* World Health Organization, *AE* adverse events, *PRR* proportional reporting ratio, *ROR* reporting odds ratio, *IC* information component, *EBGM* empirical Bayes geometric mean.

^a^All serious cardiac adverse events were identified by preferred terms included in important medical event lists from European medicines agency.

^b^Probability measured by gradient boosting machine prediction model, signal threshold > 0.5

^c^Threshold of disproportional analyses were the lower limit of 95% confidence interval (> 1 for PRR05 and ROR05; > 0 for IC05; ≥ 2 for EBGM05).

### AEs co-reported with the serious cardiovascular AEs of tisagenlecleucel

**Figure 2:**
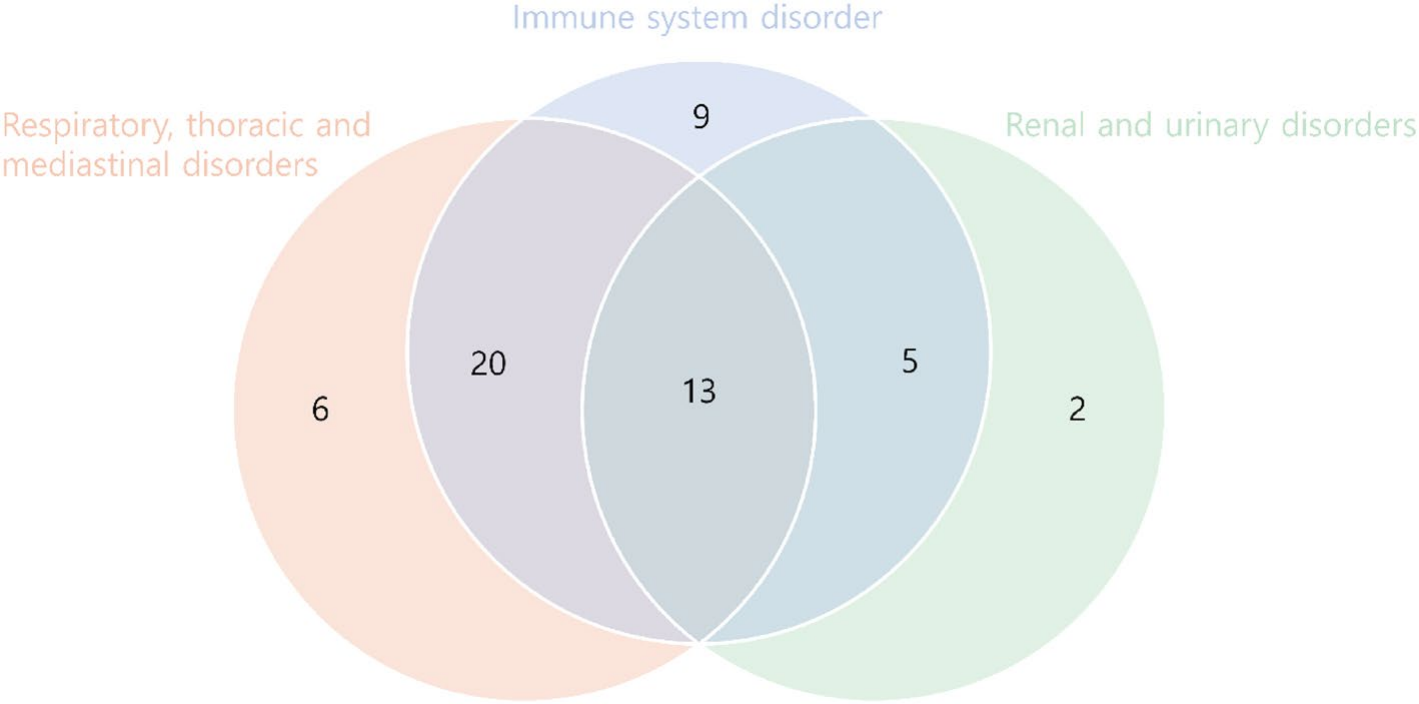
Distribution of adverse events of respiratory, immune system and renal disorders in the reports of serious cardiovascular adverse events associated with tisagenlecleucel.

Based on MedDRA system organ class (SOC), Of 72 reports on the serious cardiac AEs, 39 (54.2%) were co-reported with “respiratory, thoracic, and mediastinal disorders”; 47 (65.3%) were reported with “immune system disorders”; and 20 (27.8%) were reported with “renal and urinary disorders”. There were 13 (18.1%) reports with all three SOCs listed together with serious cardiac AEs (Fig. 2).

### Performance of signal prediction XGBoost model

**Figure 3:**
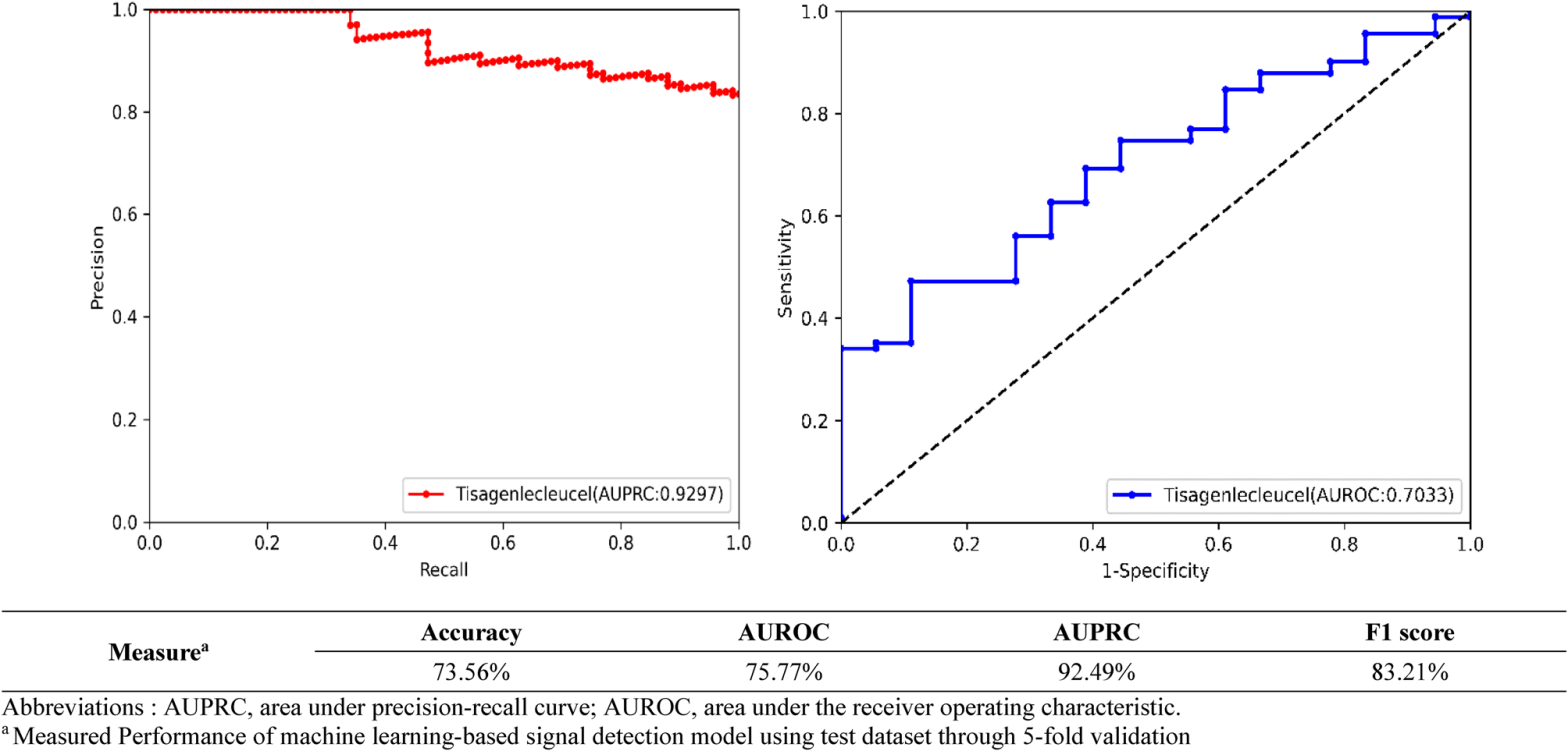
Performance of machine learning (XGBoost model) in predicting adverse events of tisagenlecleucel.

XGBoost model achieved good predictive performance in detecting AEs of tisagenlecleucel in the test dataset (AUROC 0.76) (Fig. 3). The model demonstrated superior predictive performance (accuracy 0.98; sensitivity 0.98; specificity 0.99; PPV 0.99; negative predictive value 0.98) over the disproportionality analytic methods (i.e., IC, PRR, ROR, and EBGM). AUROCs of the disproportionality analyses were 0.67 for PRR and ROR, 0.61 for IC and 0.56 for EBGM (Supplementary Table 2).

## Discussion

In this international pharmacovigilance study, we used the XGBoost-based signal prediction machine with a high statistical performance and identified six cardiovascular AEs of tisagenlecleucel. These identified safety signals of tisagenlecleucel were consistent with those described in the previous studies that reported safety signals of CAR-T products^11,13^. Notably, these events were commonly reported along with CRS, a well-known complication of CAR-T therapies.

Increase in cytokine levels in the body can lead to prolonged activation of signaling pathways such as MAPK, NF-kB, JAK-STAT3, and mTOR, which can result in secondary organ dysfunction including hepatic, renal and pulmonary disorders^28^. Furthermore, the excessive effector immune activation caused by CAR-T therapy can trigger hyperinflammation, potentially leading to immune disorders^29^. Also in our study, it was noteworthy that among the 72 cases that reported serious cardiovascular AEs, immune system disorder was the most frequently co-reported term in the MedDRA SOC terms. Considering this, the cardiac safety signals appear to have been affected by CRS.

This study utilized the most recent, largest, international database. Because of relatively short experiences in the use and small number of those treated with CAR-T therapies, there was a paucity of data on the real-world safety of this novel therapeutic. In this respect, our finding has clinical significance in that it highlights potential cardiotoxicity profile of CAR-T therapies. We have not only successfully implemented the supervised machine learning in detecting serious cardiovascular AEs of tisagenlecleucel but also demonstrated its superior performance over the traditional disproportionality analytic methods in safety signal detection. This is meaningful in that our findings are in support of machine learning-driven signal detection in replacing the traditional disproportionality analysis for routine pharmacovigilance. However, it should also be noted that the performance of supervised machine learning approach relies on a quality of an input data, which likely lead to reduced practicality and reproducibility. In this regard, rather than fully replacing the traditional analytic methods, machine learning can be used for signal refinement, filtering out false positive safety signals and capturing false negative AEs from the existing data mining methods in pharmacovigilance.

Several limitations need to be considered in interpretating our study findings. First, inherent limitations associated with the use of spontaneous reporting database need to be considered in interpreting this study’s findings. For instance, not all AEs experienced by patients who received tisagenlecleucel were recorded in VigiBase, and thus bias from underreporting is possible. Conversely, overreporting of the AEs is also possible considering that tisagenlecleucel is a relatively new product that may lead to temporary increases in the AE reporting during the early post-marketing period. Moreover, cases reported from consumers or non-healthcare professional may be missing clinically important information such as medical information and re-administration reactions. This could lead to less completeness of the database compared to the data collected from other sources. To compensate for this, we processed the data as strictly as possible within the given database with reference to guideline on GVP–Module IX–signal management^24^.

Second, there is a limitation on the construction of label data in training the machine learning model. In order to construct a label dataset, there must be a sufficient amount of product label data and relevant research conducted beforehand. However, in the case of tisagencleucel, safety data were scarce due to its relatively short period of use in the routine clinical care settings. Moreover, there may be a potential for misclassifying some of the AEs into negative controls in the label dataset, especially considering the novelty and relatively small volume of the currently available safety data of the CAR-T therapies. Such misclassification may adversely impact the performance and reproducibility of machine learning in predicting new safety signals^30^. Therefore, our findings need careful interpretation considering the available bodies of literature at the time of the study period, and the AE classification in label data is expected to change with the continued use of CAR-T therapies in the real-world setting. Third, to date, there are only few works that have conducted signal detection using machine learning approach, so there are no established gold-standard methods in constructing the training dataset^22,23^. Nevertheless, we made efforts to ensure the validity of our research by incorporating additional variables into the dataset for model training, taking into account the variables considered in existing signal detection and referencing the guideline on GVP–Module IX–signal management^24^.

In conclusion, we detected seven safety signals related to serious cardiovascular AEs that may adversely affect patient outcome after receiving tisagenlecleucel or complicate the treatment course. Our finding suggest that clinicians should be alert for these acute cardiovascular events during CAR-T therapy. Meanwhile, further studies are needed to build upon the findings from this study to explore predictors of CAR-T therapy-related cardiac complications.

### Supplementary Information

Supplementary Tables.

## Data Availability

The proposed framework implemented using SAS and Python, along with results generated in the study are available in the “Tisa-Cel Signal-Detection” repository, https://github.com/SKKUPEPV/Tisa-Cel_Signal-Detection. The data that support the findings of this study are available from Uppsala Monitoring Centre, but restrictions apply to the availability of these data, which were used under license for the current study, and so are not publicly available. Data are however available from the authors upon reasonable request and with permission of Uppsala Monitoring Centre.
