# Supplementary material for: A real-world pharmacovigilance study on cardiovascular adverse events of tisagenlecleucel using machine learning approach
Source: Sci Rep. 2024 Jun 13;14:13641. doi: 10.1038/s41598-024-64466-x (PMC11176352; doi:10.1038/s41598-024-64466-x)
Supplement: Supplementary file 1 — Supplementary Tables. [file 41598_2024_64466_MOESM1_ESM.docx]

**Supplementary table 1.** Label dataset and reference

| PT Code | PT (English) | Label | Source |  | Comments |
| --- | --- | --- | --- | --- | --- |
| 10000059 | Abdominal discomfort | 1 | tisagenlecleucel product label |  | "Abdominal pain" in Table 2 in Section 4.8 Undesirable effects |
| 10000060 | Abdominal distension | 1 | tisagenlecleucel product label |  | "Abdominal distension" in Table 2 in Section 4.8 Undesirable effects |
| 10000077 | Abdominal mass | 0 |  |  |  |
| 10000081 | Abdominal pain | 1 | tisagenlecleucel product label |  | "Abdominal pain" in Table 2 in Section 4.8 Undesirable effects |
| 10000486 | Acidosis | 1 | tisagenlecleucel product label |  | "high level of uric acid" in Section 4 Possible side effects |
| 10000636 | Activated partial thromboplastin time prolonged | U | axicabtagene ciloleucel product label |  | "Haemorrhages" in Table 2 in Section 4.8 Undesirable effects |
| 10000891 | Acute myocardial infarction | U | Literature |  | Cardiovascular events in patients treated with chimeric antigen receptor T-cell therapy for aggressive B-cell lymphoma. Haematologica. 2022 Jul 1 |
| 10001052 | Acute respiratory distress syndrome | 1 | tisagenlecleucel product label |  | "Acute respiratory distress syndrome" in Table 2 in Section 4.8 Undesirable effects |
| 10001053 | Acute respiratory failure | 1 | tisagenlecleucel product label |  | "Acute respiratory failure" in Table 2 in Section 4.8 Undesirable effects |
| 10001488 | Aggression | 0 |  |  |  |
| 10001497 | Agitation | 1 | tisagenlecleucel product label |  | "Agitation" in Table 2 in Section 4.8 Undesirable effects |
| 10001547 | Alanine aminotransferase abnormal | 1 | tisagenlecleucel product label |  | "Alanine aminotransferase increased" in Table 2 in Section 4.8 Undesirable effects |
| 10001551 | Alanine aminotransferase increased | 1 | tisagenlecleucel product label |  | "Alanine aminotransferase increased" in Table 2 in Section 4.8 Undesirable effects |
| 10001854 | Altered state of consciousness | 1 | tisagenlecleucel product label |  | "Mental status changes" in Table 2 in Section 4.8 Undesirable effects |
| 10001949 | Amnesia | 1 | tisagenlecleucel product label |  | "Memory impairment" in Table 2 in Section 4.8 Undesirable effects |
| 10002034 | Anaemia | 1 | tisagenlecleucel product label |  | "Anaemia" in Table 2 in Section 4.8 Undesirable effects |
| 10002198 | Anaphylactic reaction | 1 | tisagenlecleucel product label |  | "Anaphylactic reaction" in Section 4.8 Undesirable effects |
| 10002424 | Angioedema | 1 | tisagenlecleucel product label |  | "Oedema" in Table 2 in Section 4.8 Undesirable effects |
| 10002847 | Anuria | 1 | tisagenlecleucel product label |  | "Anuria" in Table 2 in Section 4.8 Undesirable effects |
| 10002855 | Anxiety | 1 | tisagenlecleucel product label |  | "Anxiety" in Table 2 in Section 4.8 Undesirable effects |
| 10002915 | Aortic valve incompetence | U | Literature |  | Cardiovascular events in patients treated with chimeric antigen receptor T-cell therapy for aggressive B-cell lymphoma. Haematologica. 2022 Jul 1 |
| 10002948 | Aphasia | 1 | tisagenlecleucel product label |  | "Aphasia" in Table 2 in Section 4.8 Undesirable effects |
| 10002961 | Aplasia | 0 |  |  |  |
| 10002974 | Apnoea | 0 |  |  |  |
| 10003062 | Apraxia | 1 | tisagenlecleucel product label |  | "motor dysfunction" in Table 2 in Section 4.8 Undesirable effects |
| 10003119 | Arrhythmia | U | axicabtagene ciloleucel product label |  | "Arrhythmia" in Table 3 in Section 4.8 Undesirable effects |
| 10003239 | Arthralgia | 1 | tisagenlecleucel product label |  | "Arthralgia" in Table 2 in Section 4.8 Undesirable effects |
| 10003445 | Ascites | 1 | tisagenlecleucel product label |  | "Ascites" in Table 2 in Section 4.8 Undesirable effects |
| 10003481 | Aspartate aminotransferase increased | 1 | tisagenlecleucel product label |  | "Aspartate aminotransferase increased" in Table 2 in Section 4.8 Undesirable effects |
| 10003504 | Aspiration | 1 | tisagenlecleucel product label |  | "Infections - pathogen unspecified, viral infections, bacterial infections" in Table 2 in Section 4.8 Undesirable effects |
| 10003549 | Asthenia | 1 | tisagenlecleucel product label |  | "Asthenia" in Table 2 in Section 4.8 Undesirable effects |
| 10003598 | Atelectasis | 0 |  |  |  |
| 10003658 | Atrial fibrillation | 1 | tisagenlecleucel product label |  | "Atrial fibrillation" in Table 2 in Section 4.8 Undesirable effects |
| 10003662 | Atrial flutter | U | Literature |  | Cardiovascular events in patients treated with chimeric antigen receptor T-cell therapy for aggressive B-cell lymphoma. Haematologica. 2022 Jul 1 |
| 10003988 | Back pain | 1 | tisagenlecleucel product label |  | "Back pain" in Table 2 in Section 4.8 Undesirable effects |
| 10003997 | Bacteraemia | 1 | tisagenlecleucel product label |  | "Infections - pathogen unspecified, viral infections, bacterial infections" in Table 2 in Section 4.8 Undesirable effects |
| 10005169 | Blindness | 1 | tisagenlecleucel product label |  | "Visual impairment" in Table 2 in Section 4.8 Undesirable effects |
| 10005287 | Blood albumin decreased | 1 | tisagenlecleucel product label |  | "Blood albumin decreased" in Table 2 in Section 4.8 Undesirable effects |
| 10005364 | Blood bilirubin increased | 1 | tisagenlecleucel product label |  | "Blood bilirubin increased" in Table 2 in Section 4.8 Undesirable effects |
| 10005395 | Blood calcium decreased | 1 | tisagenlecleucel product label |  | "hypocalcaemia" in Table 2 in Section 4.8 Undesirable effects |
| 10005420 | Blood chloride increased | 0 |  |  |  |
| 10005482 | Blood creatinine decreased | 1 | tisagenlecleucel product label |  | "Blood creatinine abnormal" in Table 2 in Section 4.8 in Undesirable effects |
| 10005483 | Blood creatinine increased | 1 | tisagenlecleucel product label |  | "Blood creatinine increased" in Table 2 in Section 4.8 Undesirable effects |
| 10005520 | Blood fibrinogen decreased | 1 | tisagenlecleucel product label |  | "Blood fibrinogen decreased" in Table 2 in Section 4.8 Undesirable effects |
| 10005521 | Blood fibrinogen increased | 1 | tisagenlecleucel product label |  | "Hypofibrinogenaemia" in Table 3 in Section 4.8 Undesirable effects |
| 10005557 | Blood glucose increased | 1 | tisagenlecleucel product label |  | "hyperglycaemia" in Table 2 in Section 4.8 Undesirable effects |
| 10005595 | Blood immunoglobulin G decreased | 1 | tisagenlecleucel product label |  | "Blood immunoglobulin G decreased" in Table 2 in Section 4.8 Undesirable effects |
| 10005630 | Blood lactate dehydrogenase increased | U | Literature |  | "Lactate dehydrogenase increased" in Standard-of-Care Axicabtagene Ciloleucel for Relapsed or Refractory Large B-Cell Lymphoma: Results From the US Lymphoma CAR T Consortium. J Clin Oncol. 22 Sep 2 |
| 10005635 | Blood lactic acid increased | 0 |  |  |  |
| 10005724 | Blood potassium decreased | 1 | tisagenlecleucel product label |  | "hypokalaemia" in Table 2 in Section 4.8 Undesirable effects |
| 10005728 | Blood pressure abnormal | 1 | tisagenlecleucel product label |  | "Hypertension" in Table 2 in Section 4.8 Undesirable effects |
| 10005734 | Blood pressure decreased | 1 | tisagenlecleucel product label |  | "Hypotension" in Table 2 in Section 4.8 Undesirable effects |
| 10005737 | Blood pressure diastolic decreased | 1 | tisagenlecleucel product label |  | "Hypotension" in Table 2 in Section 4.8 Undesirable effects |
| 10005750 | Blood pressure increased | 1 | tisagenlecleucel product label |  | "Hypertension" in Table 2 in Section 4.8 Undesirable effects |
| 10005758 | Blood pressure systolic decreased | 1 | tisagenlecleucel product label |  | "Hypotension" in Table 2 in Section 4.8 Undesirable effects |
| 10005760 | Blood pressure systolic increased | 1 | tisagenlecleucel product label |  | "Hypertension" in Table 2 in Section 4.8 Undesirable effects |
| 10005802 | Blood sodium decreased | 1 | tisagenlecleucel product label |  | "Hyponatraemia" in Table 2 in Section 4.8 Undesirable effects |
| 10005839 | Blood triglycerides increased | 0 |  |  |  |
| 10005850 | Blood urea decreased | 0 |  |  |  |
| 10005851 | Blood urea increased | 1 | tisagenlecleucel product label |  | "blood urine present" in Table 2 in Section 4.8 Undesirable effects |
| 10005860 | Blood uric acid decreased | 0 |  |  |  |
| 10005861 | Blood uric acid increased | 1 | tisagenlecleucel product label |  | "high level of uric acid" in Section 4 Possible side effects |
| 10005911 | Body temperature increased | 1 | tisagenlecleucel product label |  | "Influenza-like illness" in Table 2 in Section 4.8 Undesirable effects |
| 10006002 | Bone pain | 1 | tisagenlecleucel product label |  | "Bone pain" in Table 2 in Section 4.8 Undesirable effects |
| 10006093 | Bradycardia | U | brexucabtagene autoleucel |  | "Bradycardias" in Table 3 in Section 4.8 Undesirable effects |
| 10006126 | Brain herniation | 1 | tisagenlecleucel product label |  | "Stroke" in Section 4 possible side effects |
| 10006473 | Bronchopulmonary aspergillosis | 1 | tisagenlecleucel product label |  | "Infections - pathogen unspecified, viral infections, bacterial infections" in Table 2 in Section 4.8 Undesirable effects |
| 10006825 | C-reactive protein increased | 1 | tisagenlecleucel product label |  | "Cytokine release syndrome" in Table 2 in Section 4.8 Undesirable effects |
| 10007189 | Capillary disorder | U | Literature |  | Cardiovascular events in patients treated with chimeric antigen receptor T-cell therapy for aggressive B-cell lymphoma. Haematologica. 2022 Jul 1 |
| 10007196 | Capillary leak syndrome | 1 | tisagenlecleucel product label |  | "Capillary leak syndrome" in Table 2 in Section 4.8 Undesirable effects |
| 10007223 | Carbon dioxide decreased | 0 |  |  |  |
| 10007515 | Cardiac arrest | 1 | tisagenlecleucel product label |  | "Cardiac arrest" in Table 2 in Section 4.8 Undesirable effects |
| 10007554 | Cardiac failure | 1 | tisagenlecleucel product label |  | "Cardiac failure" in Table 2 in Section 4.8 Undesirable effects |
| 10007559 | Cardiac failure congestive | 1 | tisagenlecleucel product label |  | "Cardiac failure" in Table 2 in Section 4.8 Undesirable effects |
| 10007617 | Cardio-respiratory arrest | U | Literature |  | Cardiovascular events in patients treated with chimeric antigen receptor T-cell therapy for aggressive B-cell lymphoma. Haematologica. 2022 Jul 1 |
| 10007636 | Cardiomyopathy | U | lisocabtagene maraleucel |  | "Cardiomyopathy" in Table 3 in Section 4.8 Undesirable effects |
| 10007649 | Cardiovascular disorder | 1 | tisagenlecleucel product label |  | "Cardiac disorder" in Table 2 in Section 4.8 Undesirable effects |
| 10007882 | Cellulitis | 1 | tisagenlecleucel product label |  | "Infections - pathogen unspecified, viral infections, bacterial infections" in Table 2 in Section 4.8 Undesirable effects |
| 10008072 | Cerebellar syndrome | 1 | tisagenlecleucel product label |  | "Cerebellar syndrome" in Table 3 in Section 4.8 Undesirable effects |
| 10008096 | Cerebral atrophy | 0 |  |  |  |
| 10008111 | Cerebral haemorrhage | 1 | tisagenlecleucel product label |  | "Cerebral haemorrhage" in Table 2 in Section 4.8 Undesirable effects |
| 10008118 | Cerebral infarction | 1 | tisagenlecleucel product label |  | "Ischaemic cerebral infarction" in Table 2 in Section 4.8 Undesirable effects |
| 10008190 | Cerebrovascular accident | 1 | tisagenlecleucel product label |  | "Cerebral haemorrhage" in Table 2 in Section 4.8 Undesirable effects |
| 10008469 | Chest discomfort | 1 | tisagenlecleucel product label |  | "Chest pain" in Table 2 in Section 4.8 Undesirable effects |
| 10008479 | Chest pain | 1 | tisagenlecleucel product label |  | "Chest pain" in Table 2 in Section 4.8 Undesirable effects |
| 10008531 | Chills | 1 | tisagenlecleucel product label |  | "Chills" in Table 2 in Section 4.8 Undesirable effects |
| 10008635 | Cholestasis | 0 |  |  |  |
| 10009192 | Circulatory collapse | 1 | tisagenlecleucel product label |  | "Thrombosis" in Table 2 in Section 4.8 Undesirable effects |
| 10009346 | Clonus | 1 | tisagenlecleucel product label |  | "Seizure" in Table 2 in Section 4.8 Undesirable effects |
| 10009657 | Clostridium difficile colitis | 1 | tisagenlecleucel product label |  | "Infections - pathogen unspecified, viral infections, bacterial infections" in Table 2 in Section 4.8 Undesirable effects |
| 10009802 | Coagulopathy | 1 | tisagenlecleucel product label |  | "Coagulopathy" in Table 2 in Section 4.8 Undesirable effects |
| 10009887 | Colitis | U | Literature |  | "Colitis" in Intensive Care in Hematological and Oncological Patients (iCHOP) Collaborative Group. New drugs, new toxicities: severe side effects of modern targeted and immunotherapy of cancer and their management. Crit Care. 217 Apr 14 |
| 10010071 | Coma | 1 | tisagenlecleucel product label |  | "lethargy" in Table 2 in Section 4.8 Undesirable effects |
| 10010305 | Confusional state | 1 | tisagenlecleucel product label |  | "Confusional state" in Table 2 in Section 4.8 Undesirable effects |
| 10010774 | Constipation | 1 | tisagenlecleucel product label |  | "Constipation" in Table 2 in Section 4.8 Undesirable effects |
| 10011224 | Cough | 1 | tisagenlecleucel product label |  | "Cough" in Table 2 in Section 4.8 Undesirable effects |
| 10011831 | Cytomegalovirus infection | 1 | tisagenlecleucel product label |  | "Infections - pathogen unspecified, viral infections, bacterial infections" in Table 2 in Section 4.8 Undesirable effects |
| 10011906 | Death | 1 | tisagenlecleucel product label |  | "Result of cytokine syndrome" in Table 2 in Section 4.8 Undesirable effects |
| 10012174 | Dehydration | 1 | tisagenlecleucel product label |  | "Degydration" in Table 3 in Section 4.8 Undesirable effects |
| 10012218 | Delirium | 1 | tisagenlecleucel product label |  | "Delirium" in Table 2 in Section 4.8 Undesirable effects |
| 10012373 | Depressed level of consciousness | 1 | tisagenlecleucel product label |  | "Depressed level of consciousness" in Table 2 in Section 4.8 Undesirable effects |
| 10012378 | Depression | U | Literature |  | "Depression" in Patient-Reported Neuropsychiatric Outcomes of Long-Term Survivors after Chimeric Antigen Receptor T Cell Therapy. Biol Blood Marrow Transplant. 22 Jan |
| 10012735 | Diarrhoea | 1 | tisagenlecleucel product label |  | "Diarrhoea" in Table 2 in Section 4.8 Undesirable effects |
| 10013036 | Diplopia | 0 |  |  |  |
| 10013395 | Disorientation | 1 | tisagenlecleucel product label |  | "Disorientation" in Table 3 in Section 4.8 Undesirable effects |
| 10013442 | Disseminated intravascular coagulation | 1 | tisagenlecleucel product label |  | "Disseminated intravascular coagulation" in Table 2 in Section 4.8 Undesirable effects |
| 10013496 | Disturbance in attention | 1 | tisagenlecleucel product label |  | "Disturbance in attention" in Table 2 in Section 4.8 Undesirable effects |
| 10013573 | Dizziness | 1 | tisagenlecleucel product label |  | "Dizziness" in Table 2 in Section 4.8 Undesirable effects |
| 10013887 | Dysarthria | 1 | tisagenlecleucel product label |  | "Dysarthria" in Table 2 in Section 4.8 Undesirable effects |
| 10013916 | Dyskinesia | 1 | tisagenlecleucel product label |  | "Dyskinesia" in Table 2 in Section 4.8 Undesirable effects |
| 10013950 | Dysphagia | 1 | tisagenlecleucel product label |  | "Dysphagia" in Table 3 in Section 4.8 Undesirable effects |
| 10013968 | Dyspnoea | 1 | tisagenlecleucel product label |  | "Dyspnoea" in Table 2 in Section 4.8 Undesirable effects |
| 10013990 | Dysuria | 0 |  |  |  |
| 10014387 | Electrocardiogram QT prolonged | 0 |  |  |  |
| 10014408 | Electroencephalogram abnormal | 1 | tisagenlecleucel product label |  | "Atrial fibrillation" in Table 2 in Section 4.8 Undesirable effects |
| 10014418 | Electrolyte imbalance | 1 | tisagenlecleucel product label |  | "Hypercalcaemia" in Table 2 in Section 4.8 Undesirable effects |
| 10014581 | Encephalitis | 1 | tisagenlecleucel product label |  | "Infections - pathogen unspecified, viral infections, bacterial infections" in Table 2 in Section 4.8 Undesirable effects |
| 10014625 | Encephalopathy | 1 | tisagenlecleucel product label |  | "Encephalopathy" in Table 2 in Section 4.8 Undesirable effects |
| 10014909 | Enterovirus infection | 1 | tisagenlecleucel product label |  | "Infections - pathogen unspecified, viral infections, bacterial infections" in Table 2 in Section 4.8 Undesirable effects |
| 10015090 | Epistaxis | 1 | tisagenlecleucel product label |  | "Epistaxis" in Table 2 in Section 4.8 Undesirable effects |
| 10015108 | Epstein-Barr virus infection | 1 | tisagenlecleucel product label |  | "Infections - pathogen unspecified, viral infections, bacterial infections" in Table 2 in Section 4.8 Undesirable effects |
| 10015150 | Erythema | 1 | tisagenlecleucel product label |  | "Erythema" in Table 2 in Section 4.8 Undesirable effects |
| 10015943 | Eye inflammation | U | axicabtagene ciloleucel product label |  | "Cytokine release syndrome" in Table 2 in Section 4.8 Undesirable effects |
| 10015995 | Eyelid ptosis | 0 |  |  |  |
| 10016062 | Facial paralysis | 1 | tisagenlecleucel product label |  | "paralysis" in Section 4 Possible side effects |
| 10016165 | Failure to thrive | 1 | tisagenlecleucel product label |  |  |
| 10016173 | Fall | 0 |  |  |  |
| 10016256 | Fatigue | 1 | tisagenlecleucel product label |  | "Fatigue" in Table 2 in Section 4.8 Undesirable effects |
| 10016288 | Febrile neutropenia | 1 | tisagenlecleucel product label |  | "Febrile neutropenia" in Table 2 in Section 4.8 Undesirable effects |
| 10016322 | Feeling abnormal | U | axicabtagene ciloleucel product label |  | "General disorders and administration site conditions" in Table 2 in Section 4.8 Undesirable effects |
| 10016581 | Fibrin D dimer increased | 1 | tisagenlecleucel product label |  | "Fibrin D dimer increased" in Table 2 in Section 4.8 Undesirable effects |
| 10016807 | Fluid retention | 1 | tisagenlecleucel product label |  | "Fluid retention" in Table 2 in Section 4.8 Undesirable effects |
| 10016825 | Flushing | 1 | tisagenlecleucel product label |  | "Flushing" in Table 2 in Section 4.8 Undesirable effects |
| 10017412 | Full blood count abnormal | 0 |  |  |  |
| 10017413 | Full blood count decreased | 1 | tisagenlecleucel product label |  | "Lymphocyte decreased" in Table 2 in Section 4.8 Undesirable effects |
| 10017523 | Fungaemia | 1 | tisagenlecleucel product label |  | "Infections - pathogen unspecified, viral infections, bacterial infections" in Table 2 in Section 4.8 Undesirable effects |
| 10017533 | Fungal infection | 1 | tisagenlecleucel product label |  | "Fungal infection" in Table 2 in Section 4.8 Undesirable effects |
| 10017577 | Gait disturbance | 1 | tisagenlecleucel product label |  | "weakness or paralysis of limbs" in Section 4 Possible side effects |
| 10017888 | Gastroenteritis | 1 | tisagenlecleucel product label |  | "Infections - pathogen unspecified, viral infections, bacterial infections" in Table 2 in Section 4.8 Undesirable effects |
| 10017944 | Gastrointestinal disorder | 1 | tisagenlecleucel product label |  | "Gastrointestinal disorder" in Table 2 in Section 4.8 Undesirable effects |
| 10017955 | Gastrointestinal haemorrhage | 1 | tisagenlecleucel product label |  | "Gastrointestinal haemorrhage" in Table 2 in Section 4.8 Undesirable effects |
| 10017964 | Gastrointestinal infection | 1 | tisagenlecleucel product label |  | "Infections - pathogen unspecified, viral infections, bacterial infections" in Table 2 in Section 4.8 Undesirable effects |
| 10018092 | Generalised oedema | 1 | tisagenlecleucel product label |  | "Generalised oedema" in Table 2 in Section 4.8 Undesirable effects |
| 10018100 | Generalised tonic-clonic seizure | 1 | tisagenlecleucel product label |  | "Generalised tonic-clonic seizure" in Table 2 in Section 4.8 Undesirable effects |
| 10018358 | Glomerular filtration rate decreased | 1 | tisagenlecleucel product label |  | "Renal failure" in Table 2 in Section 4.8 Undesirable effects |
| 10018651 | Graft versus host disease | 1 | tisagenlecleucel product label |  | "Graft-versus-host Disease" in Table 2 in Section 4.8 Undesirable effects |
| 10018838 | Haematocrit decreased | 1 | tisagenlecleucel product label |  | "haemoglobin decreased" in Section 4.8 Undesirable effects |
| 10018867 | Haematuria | 1 | tisagenlecleucel product label |  | "Haematuria" in Table 2 in Section 4.8 Undesirable effects |
| 10018884 | Haemoglobin decreased | 1 | tisagenlecleucel product label |  | "Haemoglobin decreased" in Table 2 in Section 4.8 Undesirable effects |
| 10018985 | Haemorrhage intracranial | 1 | tisagenlecleucel product label |  | "Cerebral haemorrhage" in Table 2 in Section 4.8 Undesirable effects |
| 10019063 | Hallucination | 1 | tisagenlecleucel product label |  | "Hallucination" in Table 2 in Section 4.8 Undesirable effects |
| 10019211 | Headache | 1 | tisagenlecleucel product label |  | "Headache" in Table 2 in Section 4.8 Undesirable effects |
| 10019303 | Heart rate increased | 1 | tisagenlecleucel product label |  | "fatst heartbeat" in Section 4 Possible side effects |
| 10019465 | Hemiparesis | 1 | tisagenlecleucel product label |  | "paralysis" in Section 4 Possible side effects |
| 10019663 | Hepatic failure | U | Literature |  | "Hepatic disorders" in Chimeric antigen receptor T-cells safety: A pharmacovigilance and meta-analysis study. Am J Hematol. 2021 Sep 1 |
| 10019670 | Hepatic function abnormal | 1 | tisagenlecleucel product label |  | "Hyperbilirubinaemia" in Table 2 in Section 4.8 Undesirable effects |
| 10019842 | Hepatomegaly | 0 |  |  |  |
| 10019847 | Hepatosplenomegaly | 0 |  |  |  |
| 10019851 | Hepatotoxicity | 1 | tisagenlecleucel product label |  | "Hepatobiliary disorders" in Table 2 in Section 4.8 Undesirable effects |
| 10019948 | Herpes simplex | 1 | tisagenlecleucel product label |  | "Infections - pathogen unspecified, viral infections, bacterial infections" in Table 2 in Section 4.8 Undesirable effects |
| 10019973 | Herpes virus infection | 1 | tisagenlecleucel product label |  | "Infections - pathogen unspecified, viral infections, bacterial infections" in Table 2 in Section 4.8 Undesirable effects |
| 10020188 | HIV test positive | 1 | tisagenlecleucel product label |  | "Infections - pathogen unspecified, viral infections, bacterial infections" in Table 2 in Section 4.8 Undesirable effects |
| 10020431 | Human herpesvirus 6 infection | 1 | tisagenlecleucel product label |  | "Infections - pathogen unspecified, viral infections, bacterial infections" in Table 2 in Section 4.8 Undesirable effects |
| 10020508 | Hydrocephalus | 0 |  |  |  |
| 10020524 | Hydronephrosis | 0 |  |  |  |
| 10020575 | Hyperammonaemia | U | axicabtagene ciloleucel product label |  | "high level of uric acid" in Section 4 Possible side effects |
| 10020578 | Hyperbilirubinaemia | 1 | tisagenlecleucel product label |  | "Hyperbilirubinaemia" in Table 2 in Section 4.8 Undesirable effects |
| 10020583 | Hypercalcaemia | 1 | tisagenlecleucel product label |  | "Hypercalcaemia" in Table 2 in Section 4.8 Undesirable effects |
| 10020635 | Hyperglycaemia | 1 | tisagenlecleucel product label |  | "Hyperglycaemia" in Table 2 in Section 4.8 Undesirable effects |
| 10020642 | Hyperhidrosis | 1 | tisagenlecleucel product label |  | "Hyperhidrosis" in Table 2 in Section 4.8 Undesirable effects |
| 10020646 | Hyperkalaemia | 1 | tisagenlecleucel product label |  | "Hyperkalaemia" in Table 2 in Section 4.8 Undesirable effects |
| 10020679 | Hypernatraemia | 1 | tisagenlecleucel product label |  | "Hypernatraemia" in Table 2 in Section 4.8 Undesirable effects |
| 10020711 | Hyperphosphataemia | 1 | tisagenlecleucel product label |  | "Hyperphosphataemia" in Table 2 in Section 4.8 Undesirable effects |
| 10020772 | Hypertension | 1 | tisagenlecleucel product label |  | "Hypertension" in Table 2 in Section 4.8 Undesirable effects |
| 10020903 | Hyperuricaemia | 1 | tisagenlecleucel product label |  | "Hyperuricaemia" in Table 2 in Section 4.8 Undesirable effects |
| 10020919 | Hypervolaemia | 0 |  |  |  |
| 10020937 | Hypoaesthesia | 1 | tisagenlecleucel product label |  | "Hypoaesthesia" in Table 2 in Section 4.8 Undesirable effects |
| 10020947 | Hypocalcaemia | 1 | tisagenlecleucel product label |  | "Hypocalcaemia" in Table 2 in Section 4.8 Undesirable effects |
| 10020983 | Hypogammaglobulinaemia | 1 | tisagenlecleucel product label |  | "Hypogammaglobulinaemia" in Table 2 in Section 4.8 Undesirable effects |
| 10021015 | Hypokalaemia | 1 | tisagenlecleucel product label |  | "Hypokalaemia" in Table 2 in Section 4.8 Undesirable effects |
| 10021027 | Hypomagnesaemia | 1 | tisagenlecleucel product label |  | "Hypomagnesaemia" in Table 2 in Section 4.8 Undesirable effects |
| 10021036 | Hyponatraemia | 1 | tisagenlecleucel product label |  | "Hyponatraemia" in Table 2 in Section 4.8 Undesirable effects |
| 10021058 | Hypophosphataemia | 1 | tisagenlecleucel product label |  | "Hypophosphataemia" in Table 2 in Section 4.8 Undesirable effects |
| 10021097 | Hypotension | 1 | tisagenlecleucel product label |  | "Hypotension" in Table 2 in Section 4.8 Undesirable effects |
| 10021143 | Hypoxia | 1 | tisagenlecleucel product label |  | "Hypoxia" in Table 2 in Section 4.8 Undesirable effects |
| 10021425 | Immune system disorder | 1 | tisagenlecleucel product label |  | "Immune system disorder" in Table 2 in Section 4.8 Undesirable effects |
| 10021789 | Infection | 1 | tisagenlecleucel product label |  | "Infections - pathogen unspecified, viral infections, bacterial infections" in Table 2 in Section 4.8 Undesirable effects |
| 10022000 | Influenza | 1 | tisagenlecleucel product label |  | "Infections - pathogen unspecified, viral infections, bacterial infections" in Table 2 in Section 4.8 Undesirable effects |
| 10022437 | Insomnia | 1 | tisagenlecleucel product label |  | "Insomnia" in Table 2 in Section 4.8 Undesirable effects |
| 10022595 | International normalised ratio increased | 1 | tisagenlecleucel product label |  | "International normalised ratio increased" in Table 2 in Section 4.8 Undesirable effects |
| 10022694 | Intestinal perforation | 0 |  |  |  |
| 10022773 | Intracranial pressure increased | 1 | tisagenlecleucel product label |  | "Ischaemic cerebral infarction" in Table 2 in Section 4.8 Undesirable effects |
| 10022998 | Irritability | 1 | tisagenlecleucel product label |  | "Irritability" in Table 2 in Section 4.8 Undesirable effects |
| 10023676 | Lactic acidosis | 0 |  |  |  |
| 10024264 | Lethargy | 1 | tisagenlecleucel product label |  | "Lethargy" in Table 2 in Section 4.8 Undesirable effects |
| 10024378 | Leukocytosis | 1 | tisagenlecleucel product label |  | "Infections - pathogen unspecified, viral infections, bacterial infections" in Table 2 in Section 4.8 Undesirable effects |
| 10024382 | Leukoencephalopathy | 1 | tisagenlecleucel product label |  | "Encephalopathy" in Table 2 in Section 4.8 Undesirable effects |
| 10024384 | Leukopenia | 1 | tisagenlecleucel product label |  | "Leukopenia" in Table 2 in Section 4.8 Undesirable effects |
| 10024570 | Lip swelling | 0 |  |  |  |
| 10024670 | Liver disorder | U | Literature |  | "Hepatic disorders" in Chimeric antigen receptor T-cells safety: A pharmacovigilance and meta-analysis study. Am J Hematol. 2021 Sep 1 |
| 10024855 | Loss of consciousness | 1 | tisagenlecleucel product label |  | "lethargy" in Table 2 in Section 4.8 Undesirable effects |
| 10025082 | Lung disorder | 1 | tisagenlecleucel product label |  | "Pulmonary disorder" in Table 2 in Section 4.8 Undesirable effects |
| 10025197 | Lymphadenopathy | 1 | tisagenlecleucel product label |  | "Blood and lymphatic system disorders" in Table 2 in Section 4.8 Undesirable effects |
| 10025256 | Lymphocyte count decreased | 1 | tisagenlecleucel product label |  | "Lymphocyte count decreased" in Table 2 in Section 4.8 Undesirable effects |
| 10025258 | Lymphocyte count increased | 0 |  |  |  |
| 10025327 | Lymphopenia | 1 | tisagenlecleucel product label |  | "Lymphopenia" in Table 2 in Section 4.8 Undesirable effects |
| 10025482 | Malaise | 1 | tisagenlecleucel product label |  | "Malaise" in Table 2 in Section 4.8 Undesirable effects |
| 10026865 | Mass | 0 |  |  |  |
| 10026996 | Mean cell haemoglobin increased | 0 |  |  |  |
| 10027141 | Melaena | 1 | tisagenlecleucel product label |  | "Melaena" in Table 2 in Section 4.8 Undesirable effects |
| 10027175 | Memory impairment | 1 | tisagenlecleucel product label |  | "Memory impairment" in Table 2 in Section 4.8 Undesirable effects |
| 10027417 | Metabolic acidosis | 0 |  |  |  |
| 10027599 | Migraine | 1 | tisagenlecleucel product label |  | "Migraine" in Table 2 in Section 4.8 Undesirable effects |
| 10027878 | Monocyte count decreased | 1 | tisagenlecleucel product label |  | "Lymphocyte count decreased" in Table 2 in Section 4.8 Undesirable effects |
| 10028024 | Mouth haemorrhage | 1 | tisagenlecleucel product label |  | "Mouth haemorrhage" in Table 2 in Section 4.8 Undesirable effects |
| 10028098 | Mucormycosis | 1 | tisagenlecleucel product label |  | "Infections - pathogen unspecified, viral infections, bacterial infections" in Table 2 in Section 4.8 Undesirable effects |
| 10028116 | Mucosal inflammation | U | axicabtagene ciloleucel product label |  | "Mucosal haemorrhage" in Table 2 in Section 4.8 Undesirable effects |
| 10028330 | Muscle rigidity | U | axicabtagene ciloleucel product label |  | "Motor dysfunction" in Table 2 in Section 4.8 Undesirable effects |
| 10028334 | Muscle spasms | 1 | tisagenlecleucel product label |  | "Muscle spasms" in Table 2 in Section 4.8 Undesirable effects |
| 10028372 | Muscular weakness | 1 | tisagenlecleucel product label |  | "myopathy" in Table 2 in Section 4.8 Undesirable effects |
| 10028391 | Musculoskeletal pain | 1 | tisagenlecleucel product label |  | "Musculoskeletal pain" in Table 2 in Section 4.8 Undesirable effects |
| 10028411 | Myalgia | 1 | tisagenlecleucel product label |  | "Myalgia" in Table 2 in Section 4.8 Undesirable effects |
| 10028521 | Mydriasis | 1 | tisagenlecleucel product label |  | "Mydriasis" in Table 3 in Section 4.8 Undesirable effects |
| 10028622 | Myoclonus | 1 | tisagenlecleucel product label |  | "Myoclonus" in Table 2 in Section 4.8 Undesirable effects |
| 10028641 | Myopathy | 1 | tisagenlecleucel product label |  | "Myopathy" in Table 2 in Section 4.8 Undesirable effects |
| 10028735 | Nasal congestion | 1 | tisagenlecleucel product label |  | "Nasal congestion" in Table 2 in Section 4.8 Undesirable effects |
| 10028810 | Nasopharyngitis | 1 | tisagenlecleucel product label |  | "Infections - pathogen unspecified, viral infections, bacterial infections" in Table 2 in Section 4.8 Undesirable effects |
| 10028813 | Nausea | 1 | tisagenlecleucel product label |  | "Nausea" in Table 2 in Section 4.8 Undesirable effects |
| 10028836 | Neck pain | 1 | tisagenlecleucel product label |  | "Neck pain" in Table 2 in Section 4.8 Undesirable effects |
| 10029155 | Nephropathy toxic | U | axicabtagene ciloleucel product label |  | "Renal and urinary disorder" in Table 2 in Section 4.8 Undesirable effects |
| 10029202 | Nervous system disorder | 1 | tisagenlecleucel product label |  | "Nervous system disorder" in Table 2 in Section 4.8 Undesirable effects |
| 10029223 | Neuralgia | 1 | tisagenlecleucel product label |  | "Neuralgia" in Table 2 in Section 4.8 Undesirable effects |
| 10029331 | Neuropathy peripheral | 1 | tisagenlecleucel product label |  | "Neuropathy peripheral" in Table 2 in Section 4.8 Undesirable effects |
| 10029350 | Neurotoxicity | 1 | tisagenlecleucel product label |  | "Immune effector cell-associated neurotoxicity syndrome" in Table 2 in Section 4.8 Undesirable effects |
| 10029354 | Neutropenia | 1 | tisagenlecleucel product label |  | "Neutropenia" in Table 2 in Section 4.8 Undesirable effects |
| 10029366 | Neutrophil count decreased | 1 | tisagenlecleucel product label |  | "Neutrophil count decreased" in Table 2 in Section 4.8 Undesirable effects |
| 10029368 | Neutrophil count increased | 0 |  |  |  |
| 10030124 | Oedema peripheral | 1 | tisagenlecleucel product label |  | "Oedema peripheral" in Table 2 in Section 4.8 Undesirable effects |
| 10030302 | Oliguria | 1 | tisagenlecleucel product label |  | "Renal failure" in Table 2 in Section 4.8 Undesirable effects |
| 10031009 | Oral pain | 1 | tisagenlecleucel product label |  | "Oral pain" in Table 2 in Section 4.8 Undesirable effects |
| 10031127 | Orthostatic hypotension | 1 | tisagenlecleucel product label |  | "Orthostatic hypotension" in Table 2 in Section 4.8 Undesirable effects |
| 10033318 | Oxygen saturation decreased | 1 | tisagenlecleucel product label |  | "Cytokine release syndrome" in Table 2 in Section 4.8 Undesirable effects |
| 10033371 | Pain | 1 | tisagenlecleucel product label |  | "Pain" in Table 2 in Section 4.8 Undesirable effects |
| 10033425 | Pain in extremity | 1 | tisagenlecleucel product label |  | "Pain in extremity" in Table 2 in Section 4.8 Undesirable effects |
| 10033546 | Pallor | 0 |  |  |  |
| 10033645 | Pancreatitis | 0 |  |  |  |
| 10033661 | Pancytopenia | 1 | tisagenlecleucel product label |  | "Pancytopenia" in Table 2 in Section 4.8 Undesirable effects |
| 10033712 | Papilloedema | 0 |  |  |  |
| 10033775 | Paraesthesia | 1 | tisagenlecleucel product label |  | "Paraesthesia" in Table 2 in Section 4.8 Undesirable effects |
| 10033799 | Paralysis | 1 | tisagenlecleucel product label |  | "paralysis" in Section 4 Possible side effects |
| 10033885 | Paraparesis | 1 | tisagenlecleucel product label |  | "paralysis" in Section 4 Possible side effects |
| 10034474 | Pericardial effusion | U | Literature |  | Cardiovascular events in patients treated with chimeric antigen receptor T-cell therapy for aggressive B-cell lymphoma. Haematologica. 2022 Jul 1 |
| 10034545 | Periorbital oedema | 0 |  |  |  |
| 10034568 | Peripheral coldness | 0 |  |  |  |
| 10034827 | Pharyngeal haemorrhage | 1 | tisagenlecleucel product label |  | "Pharyngeal haemorrhage" in Table 2 in Section 4.8 Undesirable effects |
| 10034960 | Photophobia | 0 |  |  |  |
| 10035528 | Platelet count decreased | 1 | tisagenlecleucel product label |  | "Platelet count decreased" in Section 4.8 Undesirable effects |
| 10035598 | Pleural effusion | 1 | tisagenlecleucel product label |  | "Pleural effusion" in Table 2 in Section 4.8 Undesirable effects |
| 10035664 | Pneumonia | 1 | tisagenlecleucel product label |  | "Infections - pathogen unspecified, viral infections, bacterial infections" in Table 2 in Section 4.8 Undesirable effects |
| 10035669 | Pneumonia aspiration | 1 | tisagenlecleucel product label |  | "Infections - pathogen unspecified, viral infections, bacterial infections" in Table 2 in Section 4.8 Undesirable effects |
| 10035742 | Pneumonitis | 1 | tisagenlecleucel product label |  | "Infections - pathogen unspecified, viral infections, bacterial infections" in Table 2 in Section 4.8 Undesirable effects |
| 10035759 | Pneumothorax | U | axicabtagene ciloleucel product label |  | "Pulmonary" in Table 2 in Section 4.8 Undesirable effects |
| 10036018 | Pollakiuria | 0 |  |  |  |
| 10037014 | Protein total decreased | 0 |  |  |  |
| 10037063 | Prothrombin time prolonged | 1 | tisagenlecleucel product label |  | "Prothrombin time prolonged" in Table 2 in Section 4.8 Undesirable effects |
| 10037087 | Pruritus | 1 | tisagenlecleucel product label |  | "Pruritus" in Table 2 in Section 4.8 Undesirable effects |
| 10037377 | Pulmonary embolism | 1 | tisagenlecleucel product label |  | "Pulmonary embolism" in Table 2 in Section 4.8 Undesirable effects |
| 10037394 | Pulmonary haemorrhage | 1 | tisagenlecleucel product label |  | "Pulmonary haemorrhage" in Table 2 in Section 4.8 Undesirable effects |
| 10037423 | Pulmonary oedema | 1 | tisagenlecleucel product label |  | "Pulmonary oedema" in Table 2 in Section 4.8 Undesirable effects |
| 10037469 | Pulse absent | 0 |  |  |  |
| 10037515 | Pupil fixed | 0 |  |  |  |
| 10037532 | Pupillary reflex impaired | 0 |  |  |  |
| 10037538 | Pupils unequal | 0 |  |  |  |
| 10037549 | Purpura | 1 | tisagenlecleucel product label |  | "Purpura" in Table 2 in Section 4.8 Undesirable effects |
| 10037660 | Pyrexia | 1 | tisagenlecleucel product label |  | "Pyrexia" in Table 2 in Section 4.8 Undesirable effects |
| 10037844 | Rash | 1 | tisagenlecleucel product label |  | "Rash" in Table 2 in Section 4.8 Undesirable effects |
| 10037868 | Rash maculo-papular | 1 | tisagenlecleucel product label |  | "Rash maculo-papular" in Table 2 in Section 4.8 Undesirable effects |
| 10037884 | Rash pruritic | 1 | tisagenlecleucel product label |  | "Rash pruritic" in Table 2 in Section 4.8 Undesirable effects |
| 10038153 | Red blood cell count decreased | 1 | tisagenlecleucel product label |  | "haemoglobin decreased" in Table 2 in Section 4.8 Undesirable effects |
| 10038435 | Renal failure | 1 | tisagenlecleucel product label |  | "Renal failure" in Table 2 in Section 4.8 Undesirable effects |
| 10038540 | Renal tubular necrosis | 1 | tisagenlecleucel product label |  | "Renal tubular necrosis" in Table 2 in Section 4.8 Undesirable effects |
| 10038669 | Respiratory arrest | 0 |  |  |  |
| 10038683 | Respiratory disorder | 1 | tisagenlecleucel product label |  | "Acute respiratory distress syndrome" in Table 2 in Section 4.8 Undesirable effects |
| 10038687 | Respiratory distress | 1 | tisagenlecleucel product label |  | "Respiratory distress" in Table 2 in Section 4.8 Undesirable effects |
| 10038695 | Respiratory failure | 1 | tisagenlecleucel product label |  | "Respiratory failure" in Table 2 in Section 4.8 Undesirable effects |
| 10038712 | Respiratory rate increased | 1 | tisagenlecleucel product label |  | "Respiratory failure" in Table 3 in Section 4.8 Undesirable effects |
| 10039020 | Rhabdomyolysis | 0 |  |  |  |
| 10039101 | Rhinorrhoea | 0 |  |  |  |
| 10039897 | Sedation | 1 | tisagenlecleucel product label |  | "depressed level of consciousness" in Table 2 in Section 4.8 Undesirable effects |
| 10039906 | Seizure | 1 | tisagenlecleucel product label |  | "Seizure" in Table 2 in Section 4.8 Undesirable effects |
| 10040047 | Sepsis | 1 | tisagenlecleucel product label |  | "Infections - pathogen unspecified, viral infections, bacterial infections" in Table 2 in Section 4.8 Undesirable effects |
| 10040070 | Septic shock | 1 | tisagenlecleucel product label |  | "Infections - pathogen unspecified, viral infections, bacterial infections" in Table 2 in Section 4.8 Undesirable effects |
| 10040247 | Serum ferritin abnormal | 1 | tisagenlecleucel product label |  | "serum ferritin increased" in Table 2 in Section 4.8 Undesirable effects |
| 10040249 | Serum ferritin decreased | 0 |  |  |  |
| 10040250 | Serum ferritin increased | 1 | tisagenlecleucel product label |  | "Serum ferritin increased" in Table 2 in Section 4.8 Undesirable effects |
| 10040560 | Shock | 1 | tisagenlecleucel product label |  | "Haemorrhage" in Table 2 in Section 4.8 Undesirable effects (Shock haemorrhage) |
| 10040752 | Sinus tachycardia | U | Literature |  | Cardiovascular events in patients treated with chimeric antigen receptor T-cell therapy for aggressive B-cell lymphoma. Haematologica. 2022 Jul 1 |
| 10040753 | Sinusitis | 1 | tisagenlecleucel product label |  | "Infections - pathogen unspecified, viral infections, bacterial infections" in Table 2 in Section 4.8 Undesirable effects |
| 10040831 | Skin disorder | 1 | tisagenlecleucel product label |  | "Skin and subcutaneous tissue disorders" in Table 2 in Section 4.8 Undesirable effects |
| 10040882 | Skin lesion | 1 | tisagenlecleucel product label |  | "Rash" in Table 2 in Section 4.8 Undesirable effects |
| 10040984 | Sleep disorder | 1 | tisagenlecleucel product label |  | "Sleep disorder" in Table 2 in Section 4.8 Undesirable effects |
| 10041045 | Slow response to stimuli | U | axicabtagene ciloleucel product label |  | "depressed level of consciousness" in Table 2 in Section 4.8 Undesirable effects |
| 10041349 | Somnolence | 1 | tisagenlecleucel product label |  | "Somnolence" in Table 2 in Section 4.8 Undesirable effects |
| 10041466 | Speech disorder | 1 | tisagenlecleucel product label |  | "Speech disorder" in Table 2 in Section 4.8 Undesirable effects |
| 10041660 | Splenomegaly | U | Literature |  | "Splenomegaly" in Perforin-deficient CAR T cells recapitulate late-onset inflammatory toxicities observed in patients. J Clin Invest. 22 Oct 1 |
| 10041962 | Status epilepticus | 1 | tisagenlecleucel product label |  | "Status epilepticus" in Table 2 in Section 4.8 Undesirable effects |
| 10042128 | Stomatitis | 1 | tisagenlecleucel product label |  | "Stomatitis" in Table 2 in Section 4.8 Undesirable effects |
| 10042316 | Subarachnoid haemorrhage | 1 | tisagenlecleucel product label |  | "Cerebral haemorrhage" in Table 2 in Section 4.8 Undesirable effects |
| 10042361 | Subdural haematoma | U | axicabtagene ciloleucel product label |  | "Cerebral haemorrhage" in Table 2 in Section 4.8 Undesirable effects |
| 10042604 | Supraventricular tachycardia | 1 | tisagenlecleucel product label |  | "Supraventricular tachycardia" in Table 2 in Section 4.8 Undesirable effects |
| 10042674 | Swelling | 1 | tisagenlecleucel product label |  | "Swelling" in Table 2 in Section 4.8 Undesirable effects |
| 10042682 | Swelling face | 1 | tisagenlecleucel product label |  | "face oedema" in Table 2 in Section 4.8 Undesirable effects |
| 10043071 | Tachycardia | 1 | tisagenlecleucel product label |  | "Tachycardia" in Table 2 in Section 4.8 Undesirable effects |
| 10043089 | Tachypnoea | 1 | tisagenlecleucel product label |  | "Tachypnoea" in Table 2 in Section 4.8 Undesirable effects |
| 10043554 | Thrombocytopenia | 1 | tisagenlecleucel product label |  | "Thrombocytopenia" in Table 2 in Section 4.8 Undesirable effects |
| 10043607 | Thrombosis | 1 | tisagenlecleucel product label |  | "Thrombosis" in Table 2 in Section 4.8 Undesirable effects |
| 10043645 | Thrombotic microangiopathy | 1 | tisagenlecleucel product label |  | "Thrombosis" in Table 2 in Section 4.8 Undesirable effects |
| 10044221 | Toxic encephalopathy | 1 | tisagenlecleucel product label |  | "Encephalopathy" in Table 2 in Section 4.8 Undesirable effects |
| 10044565 | Tremor | 1 | tisagenlecleucel product label |  | "Tremor" in Table 2 in Section 4.8 Undesirable effects |
| 10045170 | Tumour lysis syndrome | 1 | tisagenlecleucel product label |  | "Tumour lysis syndrome" in Table 2 in Section 4.8 Undesirable effects |
| 10045555 | Unresponsive to stimuli | 1 | tisagenlecleucel product label |  | "lethargy" in Table 2 in Section 4.8 Undesirable effects |
| 10046274 | Upper gastrointestinal haemorrhage | 1 | tisagenlecleucel product label |  | "Gastrointestinal haemorrhage" in Table 2 in Section 4.8 Undesirable effects |
| 10046306 | Upper respiratory tract infection | 1 | tisagenlecleucel product label |  | "Infections - pathogen unspecified, viral infections, bacterial infections" in Table 2 in Section 4.8 Undesirable effects |
| 10046543 | Urinary incontinence | 0 |  |  |  |
| 10046571 | Urinary tract infection | 1 | tisagenlecleucel product label |  | "Infections - pathogen unspecified, viral infections, bacterial infections" in Table 2 in Section 4.8 Undesirable effects |
| 10046735 | Urticaria | 1 | tisagenlecleucel product label |  | "Rash" in Table 2 in Section 4.8 Undesirable effects |
| 10047302 | Ventricular tachycardia | 1 | tisagenlecleucel product label |  | "tachycardia" in Table 2 in Section 4.8 Undesirable effects |
| 10047461 | Viral infection | 1 | tisagenlecleucel product label |  | "Viral infection" in Table 2 in Section 4.8 Undesirable effects |
| 10047482 | Viral upper respiratory tract infection | 1 | tisagenlecleucel product label |  | "Infections - pathogen unspecified, viral infections, bacterial infections" in Table 2 in Section 4.8 Undesirable effects |
| 10047513 | Vision blurred | 1 | tisagenlecleucel product label |  | "Vision blurred" in Table 2 in Section 4.8 Undesirable effects |
| 10047571 | Visual impairment | 1 | tisagenlecleucel product label |  | "Visual impairment" in Table 2 in Section 4.8 Undesirable effects |
| 10047700 | Vomiting | 1 | tisagenlecleucel product label |  | "Vomiting" in Table 2 in Section 4.8 Undesirable effects |
| 10047895 | Weight decreased | 1 | tisagenlecleucel product label |  | "Weight decreased" in Table 2 in Section 4.8 Undesirable effects |
| 10047899 | Weight increased | 0 |  |  |  |
| 10047924 | Wheezing | 1 | tisagenlecleucel product label |  | "Difficulty breathing" in Section 4 Possible side effects |
| 10047942 | White blood cell count decreased | 1 | tisagenlecleucel product label |  | "White blood cell count decreased" in Table 2 in Section 4.8 Undesirable effects |
| 10047943 | White blood cell count increased | 1 | tisagenlecleucel product label |  | "Lymphopenia" in Table 2 in Section 4.8 Undesirable effects |
| 10048294 | Mental status changes | 1 | tisagenlecleucel product label |  | "Mental status changes" in Table 2 in Section 4.8 Undesirable effects |
| 10048610 | Cardiotoxicity | U | Literature |  | Cardiovascular events in patients treated with chimeric antigen receptor T-cell therapy for aggressive B-cell lymphoma. Haematologica. 2022 Jul 1 |
| 10048865 | Hypoacusis | 0 |  |  |  |
| 10048962 | Brain oedema | 1 | tisagenlecleucel product label |  | "cerebral oedema" in Table 2 in Section 4.8 Undesirable effects |
| 10049151 | Neutropenic sepsis | 1 | tisagenlecleucel product label |  | "Infections - pathogen unspecified, viral infections, bacterial infections" in Table 2 in Section 4.8 Undesirable effects |
| 10049199 | Hepatic cytolysis | U | axicabtagene ciloleucel product label |  | "Hepatic enzyme increased" in Table 2 in Section 4.8 Undesirable effects |
| 10049220 | C-reactive protein decreased | 0 |  |  |  |
| 10049471 | Blood phosphorus decreased | 1 | tisagenlecleucel product label |  | "Hypophosphataemia" in Table 2 in Section 4.8 Undesirable effects |
| 10049694 | Left ventricular dysfunction | 1 | tisagenlecleucel product label |  | "Left ventricular dysfunction" in Table 2 in Section 4.8 Undesirable effects |
| 10049771 | Shock haemorrhagic | 1 | tisagenlecleucel product label |  | "Haemorrhage" in Table 2 in Section 4.8 Undesirable effects |
| 10049848 | Balance disorder | 1 | tisagenlecleucel product label |  | "Ataxia" in Table 2 in Section 4.8 Undesirable effects |
| 10050528 | Ejection fraction decreased | 1 | tisagenlecleucel product label |  | "Heart failure" in Table 2 in Section 4.8 Undesirable effects |
| 10050685 | Cytokine storm | 1 | tisagenlecleucel product label |  | "Cytokine release syndrome" in Table 2 in Section 4.8 Undesirable effects |
| 10051017 | Staphylococcal bacteraemia | 1 | tisagenlecleucel product label |  | "Infections - pathogen unspecified, viral infections, bacterial infections" in Table 2 in Section 4.8 Undesirable effects |
| 10051055 | Deep vein thrombosis | 1 | tisagenlecleucel product label |  | "Deep vein thrombosis" in Table 2 in Section 4.8 Undesirable effects |
| 10051093 | Cardiopulmonary failure | U | Literature |  | Cardiovascular events in patients treated with chimeric antigen receptor T-cell therapy for aggressive B-cell lymphoma. Haematologica. 2022 Jul 1 |
| 10051125 | Hypofibrinogenaemia | 1 | tisagenlecleucel product label |  | "blood fibrinogen decreased" in Table 2 in Section 4.8 Undesirable effects |
| 10051267 | Facial paresis | 1 | tisagenlecleucel product label |  | "paralysis" in Section 4 Possible side effects |
| 10051290 | Central nervous system lesion | 1 | tisagenlecleucel product label |  | "neuropathy, "immune effector cell-associated neurotoxicity syndrome" in Table 2 in Section 4.8 Undesirable effects |
| 10051313 | B-lymphocyte count decreased | 1 | tisagenlecleucel product label |  | "Lymphocyte count decreased" in Table 2 in Section 4.8 Undesirable effects |
| 10051379 | Systemic inflammatory response syndrome | 1 | tisagenlecleucel product label |  | "Cytokine release syndrome" in Table 2 in Section 4.8 Undesirable effects |
| 10051513 | Viral sinusitis | 1 | tisagenlecleucel product label |  | "Infections - pathogen unspecified, viral infections, bacterial infections" in Table 2 in Section 4.8 Undesirable effects |
| 10051608 | Platelet count increased | 0 |  |  |  |
| 10051620 | Cytomegalovirus test positive | 1 | tisagenlecleucel product label |  | "Infections - pathogen unspecified, viral infections, bacterial infections" in Table 2 in Section 4.8 Undesirable effects |
| 10051792 | Infusion related reaction | 1 | tisagenlecleucel product label |  | "Infusion related reaction" in Table 2 in Section 4.8 Undesirable effects |
| 10051905 | Coronavirus infection | 1 | tisagenlecleucel product label |  | "Infections - pathogen unspecified, viral infections, bacterial infections" in Table 2 in Section 4.8 Undesirable effects |
| 10051910 | Enterobacter infection | 1 | tisagenlecleucel product label |  | "Infections - pathogen unspecified, viral infections, bacterial infections" in Table 2 in Section 4.8 Undesirable effects |
| 10052015 | Cytokine release syndrome | 1 | tisagenlecleucel product label |  | "Cytokine release syndrome" in Table 2 in Section 4.8 Undesirable effects |
| 10052076 | Haemodynamic instability | 1 | tisagenlecleucel product label |  | "Investigations" in Table 2 in Section 4.8 Undesirable effects |
| 10052238 | Escherichia urinary tract infection | 1 | tisagenlecleucel product label |  | "Infections - pathogen unspecified, viral infections, bacterial infections" in Table 2 in Section 4.8 Undesirable effects |
| 10052904 | Musculoskeletal stiffness | 1 | tisagenlecleucel product label |  | "weakness or paralysis of limbs" in Section 4 Possible side effects |
| 10053159 | Organ failure | 1 | tisagenlecleucel product label |  | "multiple organ dysfunction syndrome" in Table 2 in Section 4.8 Undesirable effects |
| 10053198 | Inappropriate antidiuretic hormone secretion | 0 |  |  |  |
| 10053213 | Febrile bone marrow aplasia | U | axicabtagene ciloleucel product label |  | "Lymphocyte decreased" in Table 2 in Section 4.8 Undesirable effects |
| 10053840 | Bacterial sepsis | 1 | tisagenlecleucel product label |  | "Infections - pathogen unspecified, viral infections, bacterial infections" in Table 2 in Section 4.8 Undesirable effects |
| 10053920 | Red cell distribution width increased | 0 |  |  |  |
| 10054107 | Nodule | 1 | tisagenlecleucel product label |  | "Infusion related reaction" in Table 2 in Section 4.8 Undesirable effects |
| 10054138 | Stenotrophomonas infection | 1 | tisagenlecleucel product label |  | "Infections - pathogen unspecified, viral infections, bacterial infections" in Table 2 in Section 4.8 Undesirable effects |
| 10054236 | Clostridium difficile infection | 1 | tisagenlecleucel product label |  | "Infections - pathogen unspecified, viral infections, bacterial infections" in Table 2 in Section 4.8 Undesirable effects |
| 10054265 | Alpha haemolytic streptococcal infection | 1 | tisagenlecleucel product label |  | "Infections - pathogen unspecified, viral infections, bacterial infections" in Table 2 in Section 4.8 Undesirable effects |
| 10054889 | Transaminases increased | 1 | tisagenlecleucel product label |  | "Transaminases increased" in Table 2 in Section 4.8 Undesirable effects |
| 10054938 | Cerebral disorder | 1 | tisagenlecleucel product label |  | "Cerebral infarction", "Cerebral haemorrhage" in Table 2 in Section 4.8 Undesirable effects |
| 10055181 | BK virus infection | 1 | tisagenlecleucel product label |  | "Infections - pathogen unspecified, viral infections, bacterial infections" in Table 2 in Section 4.8 Undesirable effects |
| 10055798 | Haemorrhage | 1 | tisagenlecleucel product label |  | "Haemorrhage" in Table 2 in Section 4.8 Undesirable effects |
| 10056342 | Pulmonary mass | U | Literature |  | "Pulmonary mass" in HLA Ligand Atlas: a benign reference of HLA-presented peptides to improve T-cell-based cancer immunotherapy. J Immunother Cancer. 221 Apr;9 |
| 10057107 | Blast cells present | 0 |  |  |  |
| 10057668 | Cognitive disorder | 1 | tisagenlecleucel product label |  | "Cognitive disorder" in Table 2 in Section 4.8 Undesirable effects |
| 10058080 | Staphylococcal infection | 1 | tisagenlecleucel product label |  | "Infections - pathogen unspecified, viral infections, bacterial infections" in Table 2 in Section 4.8 Undesirable effects |
| 10058151 | Pulseless electrical activity | U | Literature |  | Cardiovascular events in patients treated with chimeric antigen receptor T-cell therapy for aggressive B-cell lymphoma. Haematologica. 2022 Jul 1 |
| 10058267 | Troponin increased | 1 | tisagenlecleucel product label |  | "Cardiac failure" in Table 2 in Section 4.8 Undesirable effects |
| 10058319 | Dysgraphia | 1 | tisagenlecleucel product label |  | "disturbance in attention" in Table 2 in Section 4.8 Undesirable effects |
| 10058558 | Hypoperfusion | 1 | tisagenlecleucel product label |  | "Hypotension" in Table 2 in Section 4.8 Undesirable effects |
| 10058666 | Cytomegalovirus infection reactivation | 1 | tisagenlecleucel product label |  | "Infections - pathogen unspecified, viral infections, bacterial infections" in Table 2 in Section 4.8 Undesirable effects |
| 10058808 | Abdominal compartment syndrome | 1 | tisagenlecleucel product label |  | "Abdominal compartment syndrome" in Table 2 in Section 4.8 Undesirable effects |
| 10059024 | Gastrointestinal toxicity | U | axicabtagene ciloleucel product label |  | "Gastrointestinal disorders" in Table 2 in Section 4.8 Undesirable effects |
| 10059570 | Blood alkaline phosphatase increased | 1 | tisagenlecleucel product label |  | "Blood alkaline phosphatase increased" in Table 2 in Section 4.8 Undesirable effects |
| 10059887 | Cytogenetic analysis abnormal | 0 |  |  |  |
| 10059895 | Urine output decreased | 1 | tisagenlecleucel product label |  | "Renal failure" in Table 2 in Section 4.8 Undesirable effects |
| 10059998 | B-lymphocyte count increased | 0 |  |  |  |
| 10060795 | Hepatic enzyme increased | 1 | tisagenlecleucel product label |  | "Hepatic enzyme increased" in Table 2 in Section 4.8 Undesirable effects |
| 10060860 | Neurological symptom | 1 | tisagenlecleucel product label |  | "Nervous system disorders" in Table 2 in Section 4.8 Undesirable effects |
| 10060931 | Adenovirus infection | 1 | tisagenlecleucel product label |  | "Infections - pathogen unspecified, viral infections, bacterial infections" in Table 2 in Section 4.8 Undesirable effects |
| 10060945 | Bacterial infection | 1 | tisagenlecleucel product label |  | "Bacterial infection" in Table 2 in Section 4.8 Undesirable effects |
| 10061043 | Clostridial infection | 1 | tisagenlecleucel product label |  | "Infections - pathogen unspecified, viral infections, bacterial infections" in Table 2 in Section 4.8 Undesirable effects |
| 10061092 | Corynebacterium infection | 1 | tisagenlecleucel product label |  | "Infections - pathogen unspecified, viral infections, bacterial infections" in Table 2 in Section 4.8 Undesirable effects |
| 10061124 | Enterococcal infection | 1 | tisagenlecleucel product label |  | "Infections - pathogen unspecified, viral infections, bacterial infections" in Table 2 in Section 4.8 Undesirable effects |
| 10061126 | Escherichia infection | 1 | tisagenlecleucel product label |  | "Infections - pathogen unspecified, viral infections, bacterial infections" in Table 2 in Section 4.8 Undesirable effects |
| 10061218 | Inflammation | 1 | tisagenlecleucel product label |  | "inflammation" in Section 4 Possible side effects |
| 10061259 | Klebsiella infection | 1 | tisagenlecleucel product label |  | "Infections - pathogen unspecified, viral infections, bacterial infections" in Table 2 in Section 4.8 Undesirable effects |
| 10061273 | Malnutrition | 1 | tisagenlecleucel product label |  | "Metabolism and nutrition disorders" in Table 2 in Section 4.8 Undesirable effects |
| 10061284 | Mental disorder | 1 | tisagenlecleucel product label |  | "Psychiatric disorders" in Table 2 in Section 4.8 Undesirable effects |
| 10061296 | Motor dysfunction | 1 | tisagenlecleucel product label |  | "Motor dysfunction" in Table 2 in Section 4.8 Undesirable effects |
| 10061354 | Pneumonia fungal | 1 | tisagenlecleucel product label |  | "Infections - pathogen unspecified, viral infections, bacterial infections" in Table 2 in Section 4.8 Undesirable effects |
| 10061372 | Streptococcal infection | 1 | tisagenlecleucel product label |  | "Infections - pathogen unspecified, viral infections, bacterial infections" in Table 2 in Section 4.8 Undesirable effects |
| 10061428 | Decreased appetite | 1 | tisagenlecleucel product label |  | "Decreased appetite" in Table 2 in Section 4.8 Undesirable effects |
| 10061470 | Proteus infection | 1 | tisagenlecleucel product label |  | "Infections - pathogen unspecified, viral infections, bacterial infections" in Table 2 in Section 4.8 Undesirable effects |
| 10061471 | Pseudomonas infection | 1 | tisagenlecleucel product label |  | "Infections - pathogen unspecified, viral infections, bacterial infections" in Table 2 in Section 4.8 Undesirable effects |
| 10061494 | Rhinovirus infection | 1 | tisagenlecleucel product label |  | "Infections - pathogen unspecified, viral infections, bacterial infections" in Table 2 in Section 4.8 Undesirable effects |
| 10061512 | Serratia infection | 1 | tisagenlecleucel product label |  | "Infections - pathogen unspecified, viral infections, bacterial infections" in Table 2 in Section 4.8 Undesirable effects |
| 10061598 | Immunodeficiency | 1 | tisagenlecleucel product label |  | "Immunodeficiency" in Table 2 in Section 4.8 Undesirable effects |
| 10061603 | Respiratory syncytial virus infection | 1 | tisagenlecleucel product label |  | "Infections - pathogen unspecified, viral infections, bacterial infections" in Table 2 in Section 4.8 Undesirable effects |
| 10061728 | Bone lesion | 0 |  |  |  |
| 10061924 | Pulmonary toxicity | U | axicabtagene ciloleucel product label |  | "Pulmonary", "Pulmonary embolism", "Pulmonary haemorrhage" in Table 2 in Section 4.8 Undesirable effects |
| 10062173 | Venoocclusive disease | 1 | tisagenlecleucel product label |  | "Thrombosis" in Table 2 in Section 4.8 Undesirable effects |
| 10062237 | Renal impairment | 1 | tisagenlecleucel product label |  | "Acute kidney injury" in Table 2 in Section 4.8 Undesirable effects |
| 10062274 | Blast cell count increased | 0 |  |  |  |
| 10062352 | Respiratory tract infection | 1 | tisagenlecleucel product label |  | "Infections - pathogen unspecified, viral infections, bacterial infections" in Table 2 in Section 4.8 Undesirable effects |
| 10062959 | Neutropenic colitis | 0 |  |  |  |
| 10063322 | Interleukin level increased | 1 | tisagenlecleucel product label |  | "Cytokine release syndrome" in Table 2 in Section 4.8 Undesirable effects |
| 10063743 | Hypophagia | 1 | tisagenlecleucel product label |  | "Decreased appetite" in Table 2 in Section 4.8 Undesirable effects |
| 10064687 | Device related infection | 1 | tisagenlecleucel product label |  | "Infections - pathogen unspecified, viral infections, bacterial infections" in Table 2 in Section 4.8 Undesirable effects |
| 10065553 | Bone marrow failure | 1 | tisagenlecleucel product label |  | "Lymphopenia", "Leukopenia", "blood fibrinogen decreased" in Table 2 in Section 4.8 Undesirable effects |
| 10065973 | Iron overload | 0 |  |  |  |
| 10066260 | Acute graft versus host disease | 1 | tisagenlecleucel product label |  | "graft-versus-host disease" in Table 2 in Section 4.8 Undesirable effects |
| 10066261 | Chronic graft versus host disease | 1 | tisagenlecleucel product label |  | "graft-versus-host disease" in Table 2 in Section 4.8 Undesirable effects |
| 10066262 | Acute graft versus host disease in skin | 1 | tisagenlecleucel product label |  | "Graft-versus-host Disease" in Table 2 in Section 4.8 Undesirable effects |
| 10066263 | Acute graft versus host disease in liver | 1 | tisagenlecleucel product label |  | "graft-versus-host disease" in Table 2 in Section 4.8 Undesirable effects |
| 10066264 | Acute graft versus host disease in intestine | 1 | tisagenlecleucel product label |  | "GvHD in gastrointestinal tract" in Table 2 in Section 4.8 Undesirable effects |
| 10066274 | Cytopenia | 1 | tisagenlecleucel product label |  | "Cytopenia" in Section 4.8 Undesirable effects |
| 10068230 | Cardiorenal syndrome | U | Literature |  | Cardiovascular events in patients treated with chimeric antigen receptor T-cell therapy for aggressive B-cell lymphoma. Haematologica. 2022 Jul 1 |
| 10068306 | Gastrointestinal bacterial infection | 1 | tisagenlecleucel product label |  | "Infections - pathogen unspecified, viral infections, bacterial infections" in Table 2 in Section 4.8 Undesirable effects |
| 10068319 | Oropharyngeal pain | 1 | tisagenlecleucel product label |  | "Oropharyngeal pain" in Table 2 in Section 4.8 Undesirable effects |
| 10069049 | Gastrointestinal viral infection | 1 | tisagenlecleucel product label |  | "Infections - pathogen unspecified, viral infections, bacterial infections" in Table 2 in Section 4.8 Undesirable effects |
| 10069339 | Acute kidney injury | 1 | tisagenlecleucel product label |  | "Acute kidney injury" in Table 2 in Section 4.8 Undesirable effects |
| 10069826 | Inflammatory marker increased | 1 | tisagenlecleucel product label |  | "Cytokine release syndrome" in Table 2 in Section 4.8 Undesirable effects |
| 10070052 | Staphylococcus test positive | 1 | tisagenlecleucel product label |  | "Infections - pathogen unspecified, viral infections, bacterial infections" in Table 2 in Section 4.8 Undesirable effects |
| 10070055 | Streptococcus test positive | 1 | tisagenlecleucel product label |  | "Infections - pathogen unspecified, viral infections, bacterial infections" in Table 2 in Section 4.8 Undesirable effects |
| 10070335 | Respirovirus test positive | 1 | tisagenlecleucel product label |  | "Infections - pathogen unspecified, viral infections, bacterial infections" in Table 2 in Section 4.8 Undesirable effects |
| 10070347 | Rubulavirus test positive | 1 | tisagenlecleucel product label |  | "Infections - pathogen unspecified, viral infections, bacterial infections" in Table 2 in Section 4.8 Undesirable effects |
| 10070863 | Toxicity to various agents | 0 |  |  |  |
| 10071066 | Posterior reversible encephalopathy syndrome | U | axicabtagene ciloleucel product label |  | "Hypertension" in Table 2 in Section 4.8 Undesirable effects |
| 10071583 | Haemophagocytic lymphohistiocytosis | 1 | tisagenlecleucel product label |  | "Haemophagocytic lymphohistiocytosis" in Table 2 in Section 4.8 Undesirable effects |
| 10071662 | N-terminal prohormone brain natriuretic peptide increased | 0 |  |  |  |
| 10072859 | Human metapneumovirus test positive | 1 | tisagenlecleucel product label |  | "Infections - pathogen unspecified, viral infections, bacterial infections" in Table 2 in Section 4.8 Undesirable effects |
| 10073485 | Abdominal lymphadenopathy | 0 |  |  |  |
| 10074170 | Candida infection | 1 | tisagenlecleucel product label |  | "Infections - pathogen unspecified, viral infections, bacterial infections" in Table 2 in Section 4.8 Undesirable effects |
| 10074171 | Aspergillus infection | 1 | tisagenlecleucel product label |  | "Infections - pathogen unspecified, viral infections, bacterial infections" in Table 2 in Section 4.8 Undesirable effects |
| 10075611 | Varicella zoster virus infection | 1 | tisagenlecleucel product label |  | "Infections - pathogen unspecified, viral infections, bacterial infections" in Table 2 in Section 4.8 Undesirable effects |
| 10077361 | Multiple organ dysfunction syndrome | 1 | tisagenlecleucel product label |  | "Multiple organ dysfunction syndrome" in Table 2 in Section 4.8 Undesirable effects |
| 10077605 | Anal incontinence | 0 |  |  |  |
| 10077692 | Liver function test increased | 1 | tisagenlecleucel product label |  | "Hepatic enzyme increased" in Table 2 in Section 4.8 Undesirable effects |
| 10078589 | B-lymphocyte count abnormal | 1 | tisagenlecleucel product label |  | "Lymphocyte count decreased" in Table 2 in Section 4.8 Undesirable effects |
| 10080721 | B-cell aplasia | 1 | tisagenlecleucel product label |  | "B-cell aplasia" in Table 2 in Section 4.8 Undesirable effects |
| 10082490 | Taste disorder | 1 | tisagenlecleucel product label |  | "Taste disorder" in Table 3 in Section 4.8 Undesirable effects |
| 10083347 | Immune effector cell-associated neurotoxicity syndrome | 1 | tisagenlecleucel product label |  | "Immune effector cell-associated neurotoxicity syndrome" in Table 2 in Section 4.8 Undesirable effects |
| 10084268 | COVID-19 | 1 | tisagenlecleucel product label |  | "Infections - pathogen unspecified, viral infections, bacterial infections" in Table 2 in Section 4.8 Undesirable effects |
| 10084271 | SARS-CoV-2 test positive | 1 | tisagenlecleucel product label |  | "Infections - pathogen unspecified, viral infections, bacterial infections" in Table 2 in Section 4.8 Undesirable effects |
| 10085507 | Post-depletion B-cell recovery | U | axicabtagene ciloleucel product label |  | "Lymphocyte decreased" in Table 2 in Section 4.8 Undesirable effects |
| 10085508 | Loss of CAR T-cell persistence | 1 | tisagenlecleucel product label |  |  |

**Supplementary Table 2.** Performance comparison with traditional signal detection methods

|  | **IC025^a^** | **PRR025^b^** | **ROR025^c^** | **EBGM05^d^** | **ML** |
| --- | --- | --- | --- | --- | --- |
| **Accuracy** | 63.13% | 69.12% | 69.12% | 56.91% | 98.47% |
| **Sensitivity** | 64.19% | 70.25% | 70.25% | 57.30% | 97.63% |
| **Specificity** | 57.75% | 63.38% | 63.38% | 54.93% | 99.32% |
| **PPV** | 88.59% | 90.75% | 90.75% | 86.67% | 99.31% |
| **NPV** | 23.98% | 29.41% | 29.41% | 20.10% | 97.67% |
| **AUC** | 60.97% | 66.81% | 66.81% | 56.11% | 98.47% |

**Abbreviation:** ML, Machine Learning.

^a^ IC025 represents the lower bound of the 95% confidence interval of information component.

^b^ PRR025 represents the lower bound of the 95% confidence interval of proportional reporting ratio.

^c^ ROR025 represents the lower bound of the 95% confidence interval of reporting odd ratio.

^d^ EBGM05 represents the lower bound of 90% confidence interval of empirical bayes geometric mean.
